# Initial organization and progressive expansion of the math-responsive brain network during the first school years

**DOI:** 10.1073/pnas.2602515123

**Published:** 2026-07-02

**Authors:** Théo Morfoisse, Séverine Becuwe, Marie Palu, Cassandra Potier-Watkins, Ghislaine Dehaene-Lambertz, Stanislas Dehaene

**Affiliations:** ^a^ Chair of Experimental Cognitive Psychology, Collège de France, Paris 75005, France; ^b^ Cognitive NeuroImaging Unit, Commissariat à l’Energie atomique et aux énergies alternatives, INSERM, Université Paris-Sud, Université Paris-Saclay, NeuroSpin Center, Gif-sur-Yvette 91191, France

**Keywords:** cognitive development, mathematical cognition, longitudinal study, education, fMRI

## Abstract

The neural mechanisms by which the developing brain acquires higher mathematical concepts from elementary intuitions remain poorly understood. Through a large-scale longitudinal functional MRI study of children from preschool through first and second grade, we tracked how neural responses to mathematical and nonmathematical statements change in the first 2 y of formal schooling, and we used these data to evaluate several theories of developmental change. Before school, when listening to math statements, children already engaged an adult-like cortical network, with partial specialization for geometry. Over the first 2 y of school, we observed an overall increase in math-related activation, a small recruitment of additional neural territory, reduced activation for facts that get better known, and a small overall increase in the dimensionality of representational space. fMRI responses to individual sentences suggest that these mechanisms, particularly in left inferior frontal gyrus and bilateral intraparietal sulcus, all contribute to children’s growing mastery of mathematical concepts.

## Significance

How the child’s brain changes with schooling, as it acquires the abstract concepts of mathematics, remains unclear. By longitudinally tracking children’s brain responses to mathematical sentences from preschool through early elementary school, we show that the cortical architecture for mathematics is already in place before schooling, with early specialization for arithmetic and geometry. Schooling does not simply recruit new brain areas or prune old ones; instead, it refines existing networks through coordinated increases and decreases in brain activity and an expansion of representational space. These findings reconcile competing theories of development and reveal how conceptual knowledge emerges within a stable neural framework.

While the foundations of mathematics are intuitive and accessible to all, its scope quickly widens to include abstract, complex, and education-dependent concepts. Proto-mathematical intuitions such as approximate number and nonsymbolic geometry emerge early in life (1–4) and are shared across cultures (5–8) and a diversity of species such as monkeys (9, 10), fishes (11, 12), or even bees (13, 14), suggesting a phylogenetically ancient foundation for nonsymbolic mathematical cognition (15, 16). In contrast, formal symbolic mathematics is unique to humans (8, 17), rests on years of cultural transmission and structured education (18, 19) and remains a source of difficulty for many children (20). How do children overcome the limits of their intuitive nonverbal mathematical abilities and expand their knowledge to include symbolic mathematics, and how does the brain adapt to this learning? While the neural mechanisms of reading acquisition are increasingly understood (21), how the brain changes with math education remains largely unknown.

One prevailing theory suggests that symbolic mathematics repurpose evolutionary ancient brain circuits originally underlying proto-mathematical skills (15, 17, 22, 23). Indeed, neuroimaging studies have identified a reproducible math-responsive cortical network involving bilateral dorsal prefrontal, intraparietal, and ventrolateral inferior temporal areas in adults (15, 24, 25) which closely resembles the network already observed in infants (1, 26), children (27, 28), and nonhuman primates (16, 29). This network supports not only nonsymbolic or elementary mathematics (30–35), but also symbolic arithmetic (32, 33, 35–41), and higher-order mathematical reasoning (42–46). Even professional mathematicians continue to engage the same overall areas when processing advanced mathematical concepts (22, 42). Yet, despite this anatomical continuity, it remains unclear how the same neural circuits can evolve from supporting basic numerical and geometric intuitions to enabling the school-based acquisition of complex symbolic mathematics. Two complementary lines of research have begun to shed light on this issue. A first series of studies comparing children to adults (30–33, 36, 39, 40, 43, 47, 48) notably revealed a developmental shift from a strong reliance on frontal regions in children to greater involvement of parietal areas in adults (27, 32, 49), likely reflecting an automatization of arithmetic fact retrieval. However, by contrasting only two points of the developmental continuum, such cross-sectional studies offer limited insight into the neural changes in between. A second set of studies has focused on finer-grained developmental trajectories, either through cross-sectional comparison of children at different ages (35, 37, 38, 41, 43, 50–52), or through longitudinal designs (39, 53–55). The latter, in particular, yielded important observations such as a transient increase in hippocampal activity and hippocampal-prefrontal-parietal functional correlation as children improve their memory for arithmetic facts (54), accompanied by an increased coupling between intraparietal and inferior temporal cortices and a decoupling from the prefrontal cortex (53, 54).

While these studies have begun to uncover neural correlates of mathematical development, significant limitations remain. First, most studies have focused on basic numerical tasks, with only a few exploring more complex or educationally relevant materials (43, 45, 50, 51). Second, longitudinal brain-imaging studies remain scarce and often limited to small samples with a wide heterogeneity of ages (e.g., 7 to 14 y-olds in ref. 53). Third, the pivotal transition from preschool intuition to formal school-based instruction, is still largely unexplored (31, 35, 39, 43). For all these reasons, we still lack a clear understanding of how the brain changes when children enter school and begin learning symbolic mathematics.

To address these gaps, we conducted a longitudinal study in children, with three consecutive functional MRI scans acquired 1 y apart (n = 149 scans) from the end of preschool through first and second grade—a pivotal period of rapid cognitive and educational change. Children performed a sentence listening task (22, 42) evaluating the veracity of statements in three domains: mathematics (including arithmetic and geometry), general knowledge (GK), and social concepts. Unlike previous studies focusing on nonsymbolic numerosity or basic numerals understanding, this design targeted a broader range of mathematical vocabulary and, crucially, their composition into meaningful but possibly incorrect statements.

**Fig. 1.**
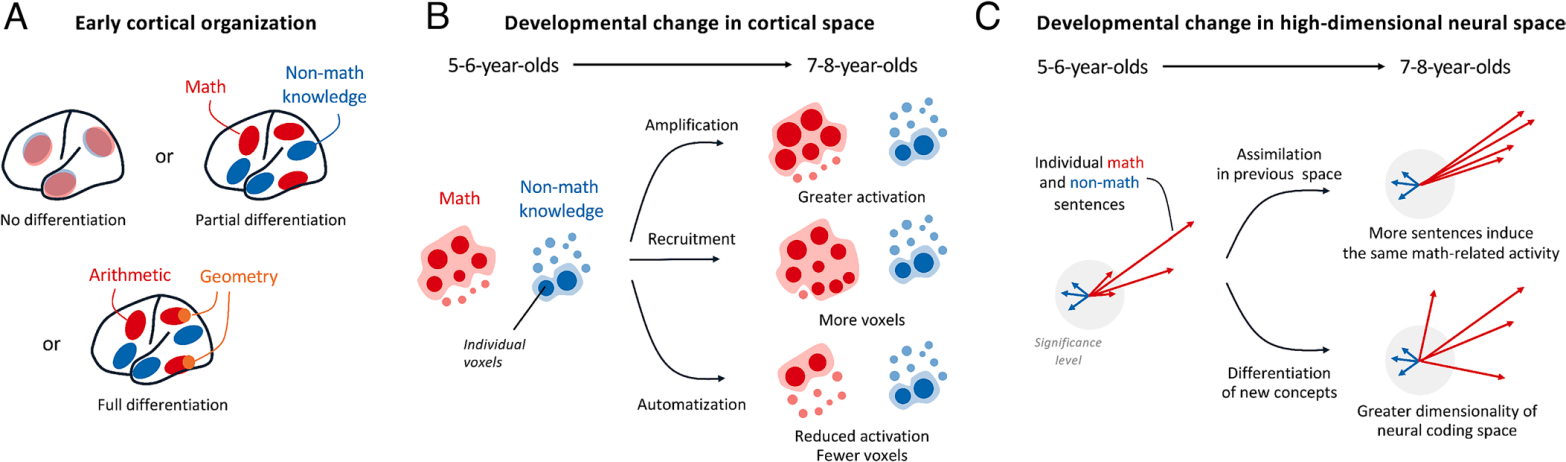
Open issues concerning the neural correlates of mathematical education. (*A*) Early cortical organization: Prior to schooling, semantic domains of math and nonmath may show minimal, partial, or full separation at the cortical level. (*B*) Developmental change in cortical space. Each circle schematizes a voxel, whose diameter indicates its activation in the contrast of math vs. nonmath statements (respectively red vs. blue). Across the first 2 y of schooling, voxels may follow i) an amplification model, where selective voxels become increasingly activated over time; ii) a recruitment model, where previously inactive or nonselective voxels are recruited for math; and iii) an automatization model, where activation to the same statements diminishes as development progresses. (*C*) Developmental change in high-dimensional neural space. Each vector represents the neural activation to each math or nonmath statement in high-dimensional neural space. With development, sentence-level activation may follow i) an assimilation model, where newly understood statements induce the same vector of math-related activity as previous ones; or ii) a differentiation model, where statements get represented by increasingly distinct neural vectors, resulting in an increased dimensionality of neural coding space.

Our design probed mathematical knowledge at three complementary levels: 1) domain-level differences, by comparing mathematical with nonmathematical statements; 2) subdomain specialization within mathematics, by contrasting arithmetic and geometry; and 3) fine-grained representational structure, by mapping neural responses to individual sentences thus characterizing the emerging space of mathematical concepts. Specifically, we first characterized the children’s mathematical network by contrasting mathematical statements with those from the two control domains. We then investigated developmental changes using domain-specific regions of interest (ROIs) defined from adult fMRI data acquired with a similar paradigm (56), allowing us to test several prominent hypotheses regarding brain changes underlying child development (57) (Fig. 1):

- **Early cortical organization** (Fig. 1*A*). Upon school entry, does the child’s brain already route verbal statements depending on their contents into adult-like circuits more responsive to math than to nonmath semantic knowledge? This hypothesis is rooted in the cortical recycling model and supported by limited prior data in preschoolers and infants (26, 31, 43). Alternatively, some of the areas might still be immature, poorly segregated, or unresponsive to composite math statements. Empiricist neo-connectionist theories (e.g., ref. 58) and parallels between human development and learning in large-language models (e.g., ref. 59) should predict that a long exposure to verbal inputs is required before a segregation of math and nonmath sentences emerges. As a finer test, we contrasted arithmetic and geometry, which also exhibit a partial segregation in adults (42, 60), to examine whether, at this age, the math-responsive network is still functionally unified, or already fully differentiated into specialized subnetworks.
- **Developmental change in cortical space** (Fig. 1*B*). How does cortical activity in the math network evolve during development? Does it involve an activation increase, as expected if the recycled areas become increasingly responsive to words and symbols (42); a decrease, if neural representations become sparser, more sharply tuned (61), and/or mobilize fewer cognitive resources (57); or both, i.e., a prefrontal decrease and a parietal increase due to greater reliance on automatized posterior circuits, as suggested by several previous experiments (27, 32, 53, 54)? If an increase is observed, does it merely involve a strengthening of existing neural responses, as observed when monkeys were trained for numerosity (62) or an expansion into new cortical territory, as observed in the case of reading (21, 63)? And can the effects of age, school exposure, and individual proficiency on these changes be separated?
- **Developmental change in high-dimensional neural space** (Fig. 1*C*). Assuming that words, and sentences are represented as neural vectors embedded in a high-dimensional manifold (64–67), how does this manifold change as new concepts are added to the semantic lexicon? Beyond conventional subject-level analyses, we adopted a sentence-centered approach that treats each sentence as a unit of analysis, allowing us to quantify the dimensionality of their neural representational space. We hypothesized that dimensionality would increase with mathematical development, reflecting the progressive differentiation of a growing number of conceptual expressions within a shared neural network (68). Alternatively, dimensionality might remain stable, or even decrease, if newly acquired concepts become aligned with preexisting neural representations.

## Results

### Behavior.

Children performed significantly above chance across all conditions, regardless of the testing period (*P* ≤ 0.001, *SI Appendix*, Fig. S1 *A* and *B*). Binomial mixed-model regression, with condition and age as fixed effects, and participant as a random effect revealed that children were significantly more accurate in judging the truth of general knowledge (GK; z = −4.08, *P* < 0.001) and social sentences (z = −5.29, *P* < 0.001) compared to mathematical ones, and that errors decreased with age across all three conditions (z = −3.91, *P* <0.001), with no significant interactions between age and condition (*SI Appendix*, Table S1). Moreover, errors for arithmetic and geometric sentences also decreased over time (respectively; z = −2.65, *P* = 0.008; z = −3.50, *P* < 0.001), with this difference falling short of significance (z = 1.95, *P* = 0.051). Similar mixed-model regressions on reaction times, revealed that children responded more quickly to social (t = −6.29, *P* < 0.001) and GK (t = −6.63, *P* < 0.001) sentences than to mathematical ones (*SI Appendix*, Table S2). No significant effect of age on reaction times was observed (t = 0.87, *P* = 0.38), but a significant interaction indicated that reaction times for social sentences decreased more with age than those for mathematical sentences (t = −2.13, *P* = 0.033) (*SI Appendix*, Fig. S1*C*).

### fMRI Characterization of the Math-Responsive Network in 5- to 8-y-olds.

**Fig. 2.**
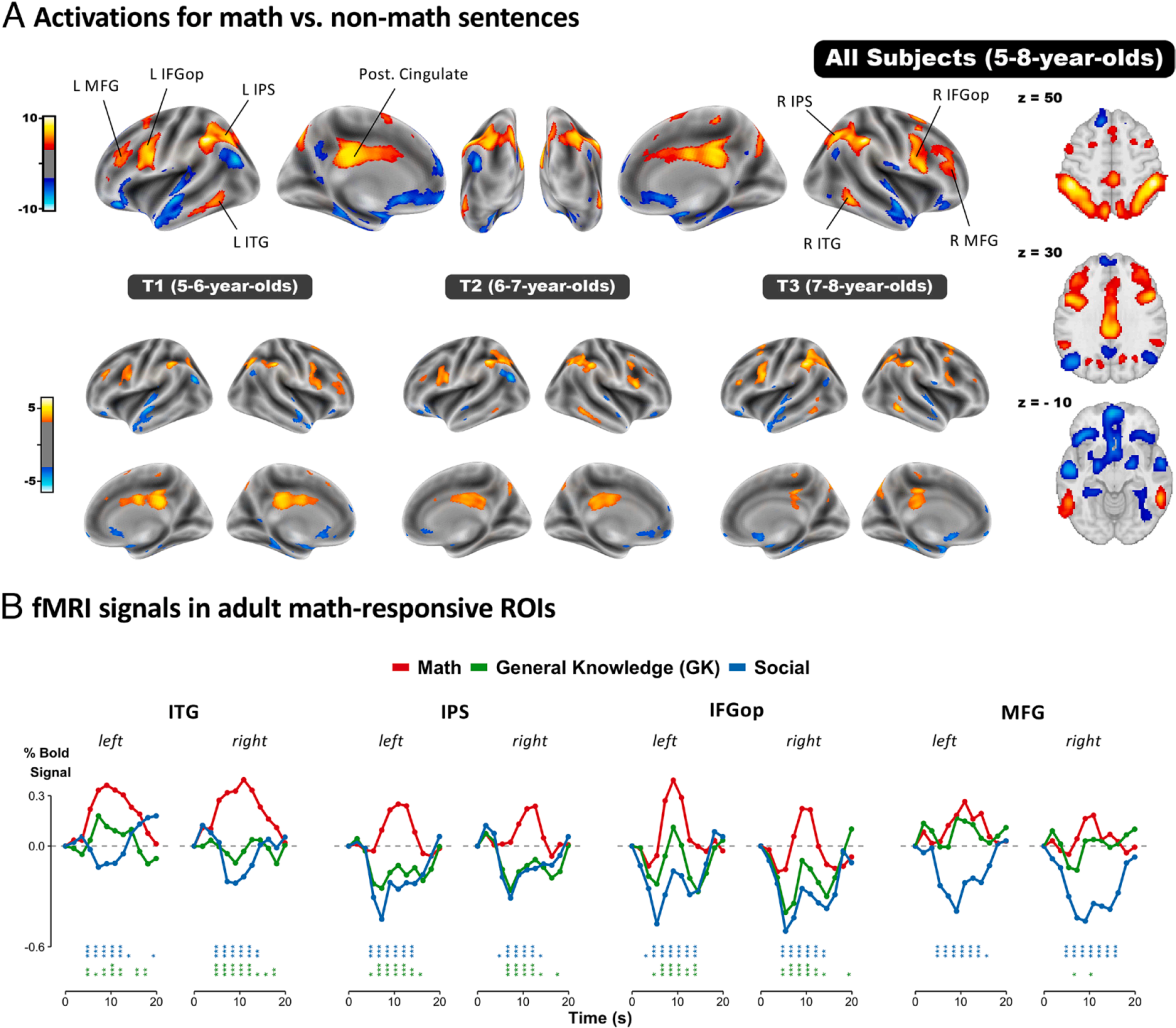
An adult-like math-responsive network in young children. (*A*) fMRI contrast between math and nonmath sentences, based on a whole-brain group analysis. The top row shows the joint analysis of all data, while bottom plots show separately the first fMRI (T1, 5 to 6-y-olds), second fMRI (T2, 6 to 7-y-olds), and third fMRI (T3, 7 to 8-y-olds). Voxel-wise *P* < 0.001, FDR-corrected α < 0.05. (*B*) Time course of average fMRI signals in math-related ROIs extracted from an adult cohort using a similar paradigm (56). Stars indicate the significance of independent mixed-model regressions conducted at each time step (TR), with condition as fixed effect and participant as random effect (•*P* < 0.1; *P < 0.05; **P < 0.01; ***P < 0.001). Blue stars: Math vs. Social. Green stars: Math vs. GK.

Actively listening to mathematical statements, as opposed to nonmath sentences (GK and Social) activated a network of bilateral brain regions in 5- to 8-y-old children, including the intraparietal sulci (IPS), inferior temporal gyri (ITG), inferior frontal gyri (IFG), middle frontal gyri (MFG), and posterior cingulate gyri (Fig. 2*A*). Importantly, this math-responsive network was identified at all three ages tested (Fig. 2*A*, bottom row). Restricting the analysis to correct trials or controlling for trial-by-trial reaction time yielded virtually identical results (*SI Appendix*, Fig. S3). As in adults, there was a double dissociation with other brain areas showing a greater response to social sentences, including the bilateral temporal and angular gyri, left inferior frontal gyrus, medial frontal gyrus, and precuneus (*SI Appendix*, Fig. S5). For direct comparison, *SI Appendix*, Fig. S4 *A* and *B* shows the math-responsive and social-responsive brain regions identified in an adult cohort using a similar paradigm (56).

From these adult activation maps, we defined eight math-responsive ROIs, specifically the left and right ITG, IPS, IFGop, and MFG (*Materials and Methods*) and computed the fMRI time course signal for all three conditions within each of these regions for each child (Fig. 2*B*). Linear mixed-model regressions performed at each time step (TR), revealed that math sentences elicited significantly stronger responses compared to social sentences in all regions, with differences emerging around 5 to 7 s after sentence onset (*P* < 0.001 and t > 4 in all regions for TR = 4). A similar divergence was observed for math vs. GK sentences, in all but two regions—the left and right MFG. Importantly, similar results were observed when we restricted the analysis to preschoolers or children within 1 mo of entering first grade (*SI Appendix*, Fig. S6).

### Dissociation Between Geometry and Arithmetic.

**Fig. 3.**
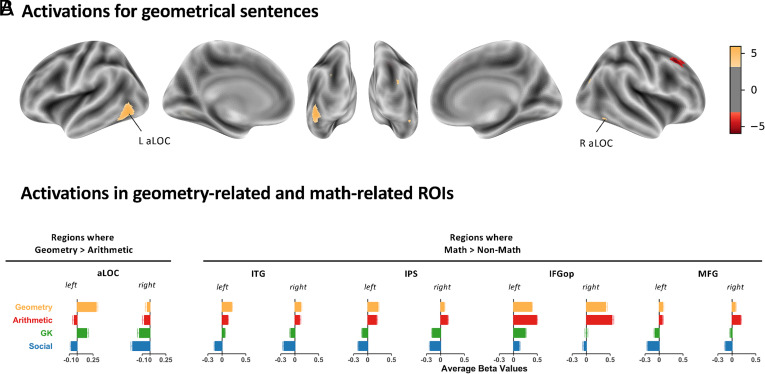
Dissociation between geometry and arithmetic. (*A*) fMRI contrast between geometric and arithmetic sentences across all sessions. Vowel-wise threshold *P* < 0.001, false discovery rate (FDR)–corrected α < 0.05. (*B*) Average β-values within geometric-related ROIs (left and right aLOC) and in math-related ROIs defined in adults (56).

Actively listening to geometric statements as opposed to arithmetic sentences selectively activated a single region—the left anterior lateral occipital cortex (aLOC) at the posterior border of the left inferior temporal gyrus (Fig. 3*A*). Based on adults’ activation maps (56), we defined two geometry-responsive ROIs (*SI Appendix*, Fig. S4*C*) and compared children’s β-values across the four conditions (geometry, arithmetic, GK, social, Fig. 3*B*). Linear mixed-model regressions confirmed that the left aLOC was significantly more activated by geometric than by either arithmetic [t(311.71) = 6.17, *P*_FDR_ < 0.001], GK [t(311.71) = 2.63, *P*_FDR_ = 0.013), or social statements [t(311.71) = 6.95, *P*_FDR_ < 0.001]. In contrast, the right aLOC showed significantly higher activation for geometry only when compared to social sentences [t(310.69) = 3.19, *P*_FDR_ = 0.004], but not in comparison to arithmetic [t(310.69) = 0.61, *P*_FDR_ = 0.54], or GK [t(310.69) = 1.72, *P*_FDR_ = 0.10].

Despite this local difference, arithmetic and geometric responses overlapped in a core common math-responsive network (Fig. 3*B* and *SI Appendix*, Fig. S7*A*). Similar mixed-model and Bayesian regressions conducted within the eight math-responsive ROIs revealed no significant differences between geometry and arithmetic after FDR correction (*SI Appendix*, Table S3). Moreover, an anterior–posterior dissociation emerged within both IPS, with anterior sections responding more to geometry and posterior ones to arithmetic (*SI Appendix*, Fig. S7*B*). Mixed-model regressions with y-coordinates as fixed effect and participant as random effect, confirmed a significant decreased of Δβ-values ($\beta_{\mathrm{Arithmetic}}-\beta_{\mathrm{Geometry}}$) along the y-axis (t = −6.40, *P* < 0.001 in the left IPS; t = −4.96, *P* < 0.001 in the right IPS).

### Development of the Math-Responsive Network.

#### Age-related increases in mathematical selectivity.

**Fig. 4.**
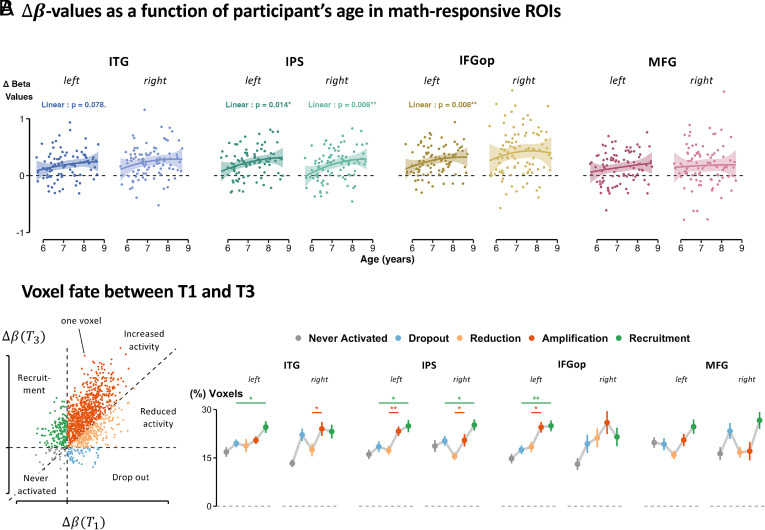
Development of the main areas of the math-responsive network. (*A*) Δβ-values ($\beta_{\mathrm{Math}}-\beta_{Non-Math}$) as a function of children’s age, within each math-responsive ROI. Mixed-model regressions were conducted within each ROI, with age as fixed effect and participant as random effect (•*P* < 0.1; *P < 0.05; **P < 0.01; ***P < 0.001). (*B*) Voxelwise math-specificity changes in children scanned at both T1 and T3 (N = 26). (*Left*) Model of voxel-activation changes. Each voxel was categorized into five categories based on its activation at T1 and T3: never activated voxels, dropout voxels, reduced voxels, amplified voxels, or finally recruited voxels. (*Right*) For each participant, we computed the percentage of voxels in each of the five categories. We reported the group averages for each of the math-responsive ROI. Linear regressions compared amplified vs. reduced voxels and recruited vs. dropout voxels (*P < 0.05; **P < 0.01; ***P < 0.001).

To track the development of the math-responsive brain network, we examined how mathematical selectivity evolves as a function of age in the eight adult math-responsive ROIs (Fig. 4*A*). Mathematical selectivity was defined as the difference (Δβ-values) between the activation elicited by math sentences and the mean activation elicited by nonmath sentences. Linear mixed-model regression revealed significant age-related increases in math selectivity in three regions: both IPS [left: t(72.06) = 2.52, *P* = 0.014, *P*_FDR_ = 0.037; right: t(62.80) = 2.83, *P* = 0.006, pFDR = 0.032], and the left IFGop [t(59.45) = 2.73, *P* = 0.008, *P*_FDR_ = 0.032] (*SI Appendix*, Table S4). When hemisphere was entered as an additional factor within regression, no significant Age × hemisphere interaction was observed (all t < 1), indicating comparable developmental trajectories across hemispheres. Similar analysis comparing geometry-related activations against all other conditions (arithmetic, GK, and social) in left and right aLOC, found no significant relationship with age (t < 1 in both aLOC). Control analyses including head motion and temporal signal-to-noise (tSNR) as covariates confirmed that the age-related changed in Δβ-values reflected genuine developmental effects rather than differences in data quality (*SI Appendix*, Tables S5 and S6, SOM). Finally, analyses were repeated using child-derived ROIs, yielding similar results including consistent age-related increases in mathematical selectivity in parietal and temporal regions (*SI Appendix*, Fig. S8). Minor differences in prefrontal regions were observed, likely due to differences in ROI parcellation rather than substantive changes in developmental trajectories.

#### Age, schooling, or numeracy?

While we used age as a proxy for child development so far, other factors such as schooling (number of days since school entry) and math ability (as measured by a numeracy screener prior to each fMRI session) may be a better predictor of developmental change in brain activation patterns. Using similar mixed-model regressions with these other variables, we observed that, for example, mathematical selectivity (Δβ-values) was significantly predicted by children’s numeracy screener scores, in four of the mathematical ROIs (*SI Appendix*, Fig. S9*A*): left IFGop [t(72.08) = 3.06, *P* = 0.003, pFDR = 0.024), both IPS [left: t(85.92) = 2.19, *P* = 0.031, *P*_FDR_ = 0.062; right: t(73.66) = 2.77, *P* = 0.007, *P*_FDR_ = 0.028], and left ITG [t(76.36) = 2.34, *P* = 0.028, *P*_FDR_ = 0.062] (*SI Appendix*, Table S7). Note however that all developmental measures were highly intercorrelated (e.g., r = 0.92 between schooling and age, r = 0.82 between age and math ability, and r = 0.77 between schooling and math ability; full correlation matrix in *SI Appendix*, Fig. S9*B*). In an effort to disentangle their contributions, we compared mixed-effects models including either schooling, chronological age, or math ability as the sole fixed predictor (participant as a random effect; *SI Appendix*, Fig. S9*C*) and estimated Akaike model weights to quantify how well each model explained the ∆β-values. Mathematical ability emerged as the strongest predictor in the left ITG (63%) and left IFGop (59%), whereas in bilateral IPS, model probabilities were more evenly distributed. In the remaining four regions, none of the predictors showed significant effects (all *P* > 0.05).

#### Changes underlying the increase in math-related activation.

What drives the age-related increase in mathematical selectivity observed in certain brain regions—an increase in the maximal activation of the same voxels, or the recruitment of additional cortex (Fig. 1*B*)? To investigate this, we tracked the fate of individual voxels across development. We capitalized on the longitudinal aspect of our data and examined the activations of participants who performed both T1 and T3 sessions (N = 26). Within each ROI, we categorized voxels into five categories based on their math-selective activation ($\Delta \beta =\beta_{\mathrm{Math}}-\beta_{Non-Math}$) at T1 and T3 (Fig. 4*B*): never activated voxels ($\Delta \beta \left(T_{3}\right)<0,and\;\Delta \beta \left(T_{1}\right)<0$), dropout voxels ($\Delta \beta \left(T_{3}\right)<0,and\;\Delta \beta \left(T_{1}\right)>0$), recruited voxels ($\Delta \beta \left(T_{3}\right)>0,and\;\Delta \beta \left(T_{1}\right)<0$) and finally, within the voxels activated in both periods ($\Delta \beta \left(T_{1}\right)>0$ and $\Delta \beta \left(T_{3}\right)>0$), those whose activity was reduced ($\Delta \beta \left(T_{3}\right)<\Delta \beta \left(T_{1}\right)$) or amplified ($\Delta \beta \left(T_{3}\right)>\Delta \beta \left(T_{1}\right)$). For each participant, we computed the percentage of voxels in each of these five conditions.

If the math-related signals were stable over development, the distribution of voxels responses in Fig. 4*B* should be symmetric around the diagonal $\Delta \beta \left(T_{1}\right)=\Delta \beta \left(T_{3}\right)$. This null hypothesis was clearly rejected due to both amplification and, to a lesser degree, recruitment. Indeed, the percentages of amplified voxels was significantly higher than the percentage of reduced voxels in the left IFGop [t(25) = 2.62, *P* = 0.01, *P*_FDR_ = 0.046], both IPS [left: t(25) = 2.92, *P* = 0.005, *P*_FDR_ = 0.042; right: t(25) = 2.18, *P* = 0.034, *P*_FDR_ = 0.068] and the right ITG [t(25) = 2.22, *P* = 0.031, *P*_FDR_ = 0.068] (Fig. 4*B* and *SI Appendix*, Table S8). We also found modest evidence for recruitment: in left IFGop, left ITG, and bilateral IPS, the percentage of recruited voxels exceeded that of dropout voxels, although these comparisons did not survive FDR correction, except for the left IFGop [t(25) = 3.45, *P* = 0.0012, *P*_FDR_ = 0.009; *SI Appendix*, Table S9). Taken together, these findings suggest that age-related increases in math responses are driven both by enhanced responses within the already-active voxels, and the recruitment of new voxel, although the latter effect seems less pronounced.

### Sentence-Level Analysis.

#### Improved comprehension correlates with decreased activation.

While we observed rapid changes in the math-responsive network between ages 5 and 8, it is unlikely that all mathematical statements engage these regions uniformly. More complex sentences may recruit these regions only in older children who possess the necessary conceptual understanding. Conversely, simpler statements may evoke stronger activation in younger children, while in older children, their comprehension may have become sufficiently automated to require fewer neural resources (automatization model in Fig. 1*B*).

**Fig. 5.**
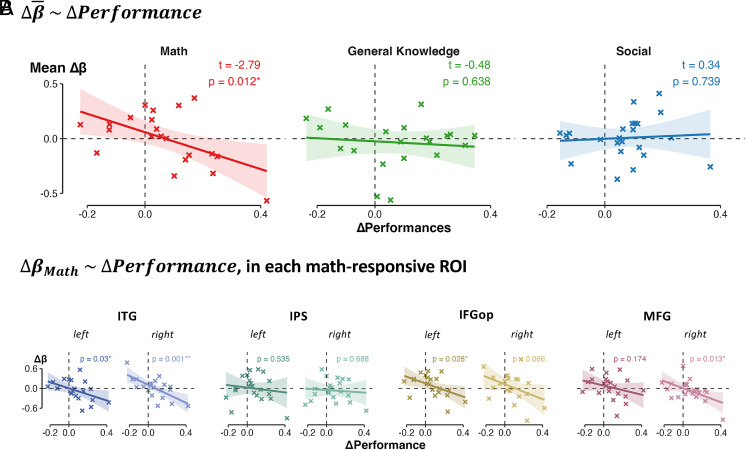
Improved comprehension leads to reduced activation for mathematical sentences in math ROIs. Only children scanned both at T1 and T3 were included here. (*A*) Mean Δβ-values ($\beta \left(T_{3}\right)-\beta \left(T_{1}\right)$) for each condition, averaged across children and math-responsive ROIs, as a function of changes in performance (*P < 0.05; **P < 0.01; ***P < 0.001). (*B*) Mean Δβ-values for math sentences and within each ROI, averaged across children, as a function of changes in performance (*P < 0.05; **P < 0.01; ***P < 0.001).

To test these possibilities and examine how changes in brain activation were related to changes in sentence comprehension, we focused our analysis on the sentences that were heard twice by the same children at both T1 and T3. For each sentence of the three domains and each child, we computed the change in brain activation (Δβ-values) in both math- and social-related ROIs, by subtracting the β-values at T3 from those at T1. For each sentence, we also computed the average behavioral performance across children at both T1 and T3 and used the difference (ΔPerf) as a proxy for change in comprehension. Across mathematical sentences, Δβ-values, averaged across children and math ROIs, decreased significantly as ΔPerf increased [t(19) = −2.79, *P* = 0.012, *P*_FDR_ = 0.036]. This was not the case for GK [t(19) < 1] or social sentences [t(21) < 1] (Fig. 5*A*). This effect was significantly stronger for math compared to social sentences [t(59) = 2.03, *P* = 0.047] and marginally greater compared to GK ones [t(59) = 1.72, *P* = 0.09]. Importantly, when performing the same analysis but within social-related ROIs, no significant relationships emerged [Math: t(19) = 1.45, *P* = 0.16, *P*_FDR_ = 0.49; GK: t(19) < 1; Social: t(21) < 1] (*SI Appendix*, Fig. S10*A*). This pattern points to a robust relationship between improved comprehension and decreased activation within math-specific regions: the better a mathematical sentence was understood over time, the less activation it elicited in the math-specific regions.

Examining each of the eight math ROIs separately (Fig. 5*B*), we observed that all regions, apart from left and right IPS, showed this pattern, with significant decreases in four regions (left and right ITG, left IFGop, and right MFG, *SI Appendix*, Table S10, although only the right ITG survived FDR correction). In contrast, none of the social regions showed significant decreases in activation–performance relationships for mathematical sentences (all *P* > 0.05, except for left TP but with t > 0; *SI Appendix*, Fig. S10*B* and Table S11).

#### An increase in dimensionality in math-related ROIs.

**Fig. 6.**
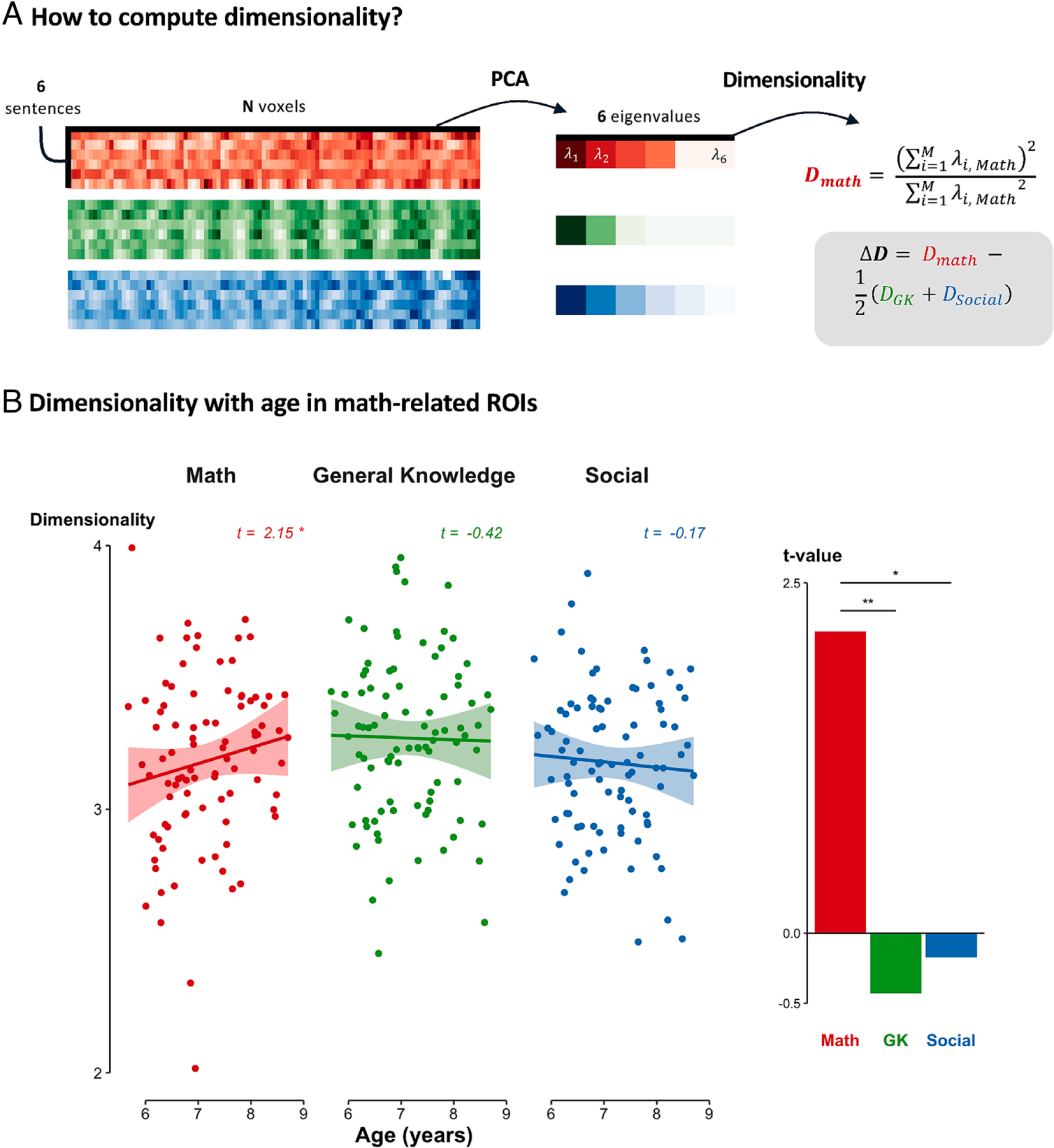
A slight increase in the differentiation of neural space for mathematical concepts. (*A*) For each run performed by each child, for each condition (Math, GK, Social), and within each ROI, we performed a principal component analysis (PCA). Eigenvalues were used to compute the intrinsical dimensionality (ID), reflecting how many dimensions represent the activation patterns. (*B*) Dimensionality values—averaged across ROI—as a function of age, in each condition. Mixed model regressions were conducted, using age as fixed effect, and including random intercepts and slopes for age per participant, and a random intercept for ROI (*P < 0.05; **P < 0.01; ***P < 0.001).

Finally, to go beyond mere activation changes and investigate *how* information is represented at the neural level, we studied developmental change in high-dimensional neural space (Fig. 1*C*). We measured their intrinsic dimensionality (ID), a quantitative measure of how many independent vector directions in cortical space are required to represent a set of distinct sentences. ID was calculated for each child, condition, and each set of ROIs (in both math- and social- related ROIs) (Fig. 6*A*). To assess how neural dimensionality evolves with age within each set of ROIs, we performed similar analyses as before but given that each participant contributed multiple measurements across brain regions and age points, the model included random intercepts and slopes for age per participant and a random intercept for ROI.

In math-related ROIs, we observed a small but significant age-related increase in dimensionality for math sentences [t(29.67) = 2.15, *P* = 0.040], and a nonsignificant decrease for GK and social sentences (both t < 1, Fig. 6*B*). In social-related ROIs, no significant age-related changes were observed for any condition [Math: t(29.20) = 1.52, *P* = 0.14; GK and Social: t < 1] (*SI Appendix*, Fig. S11*A*). When condition was added as a predictor, a significant age × condition interaction emerged in math-related ROIs between math and GK sentences (t = −3.03, *P* = 0.002) and math and social sentences (t = −2.46, *P* = 0.014), but not in social-related ROIs (Math vs. GK: t = −1.25, *P* = 0.21; Math vs. Social: t = −1.52, *P* = 0.13). These results suggest that, during the first 2 y of schooling, math-responsive brain regions exploit a higher-dimensional neural space to represent mathematical information, while social-responsive brain regions did not show such developmental change in our age range.

We then performed linear regression in each of the eight math-related ROIs individually (*SI Appendix*, Fig. S11*B*). Although most regions showed a positive age-related increase in ΔDimensionality (i.e., differences between math dimensionality and nonmath dimensionality), only two—the left ITG and right MFG—showed a statistically significant effect [left ITG: t(89) = 2.20, *P* = 0.029, right MFG: t(89) = 2.48, *P* = 0.014] which did not survive FDR correction (*P*_FDR_ = 0.12 and 0.11 respectively) (*SI Appendix*, Table S12).

## Discussion

**Fig. 7.**
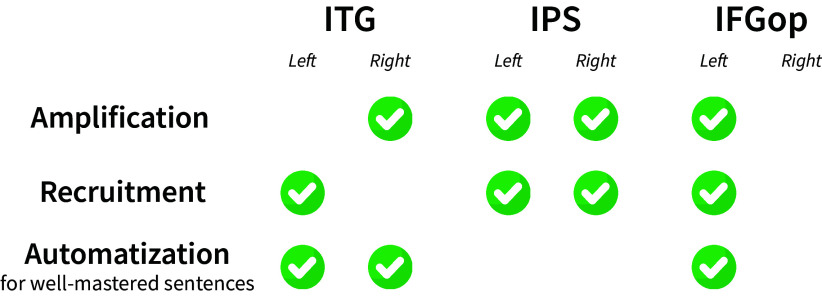
Summary of developmental changes in cortical space. Green crosses indicate significant changes.

We investigated fMRI responses in 6- to 9-y-old children using a sentence-listening paradigm previously used only in adults (22, 24, 42). Our findings provide compelling evidence that the neural architecture underlying mathematical cognition is already well-established prior to schooling, including a partial dissociation between arithmetic and geometry. Over the first 2 y of school, developmental changes include: increased peak activation for math relative to nonmath in bilateral IPS, left IFGop, and right ITG; an expansion of cortical territory in bilateral IPS, left IFGop, and left ITG; activation decreased when sentences meaning became better mastered, in bilateral ITG, left IFGop, and right MFG; and a slight increase in the dimensionality of neural space encoding math sentences (Fig. 7). We now discuss these points in turn.

Our study extends to young children, including preschoolers, the existence of a network of brain regions selectively engaged when actively processing mathematical sentences compared to nonmath ones (22, 42). It includes a well-documented fronto-parietal network but also extends to regions less frequently emphasized in the literature, including the bilateral inferior temporal gyrus and bilateral posterior cingulate (Fig. 2). This early mathematical brain network closely mirrors the well-characterized math-responsive network in adults (15, 16, 24, 25) and children (35, 47, 69). The present findings indicate that this network can already be engaged, not only by concrete mathematical objects such as sets of dots, but also by purely verbal symbols. Thus, even at the very beginning of schooling, mathematical language is not only understood but also functionally integrated and capable of recruiting a math-responsive cortical network. In agreement with the triple-code model of number processing (70), bilateral IPS appears as a semantic convergence zone capable of activating from purely verbal inputs, and now seen to do so 1) since preschool 2) for both arithmetic and geometry, and 3) in tight coordination with bilateral ITG, IFG, and MFG.

While several studies have probed the neural bases of symbolic vs. nonsymbolic numerical cognition (32, 33, 35, 69) relatively few directly compared different mathematical subfields (71). A recent study showed broad overlap in brain activation when children compared numbers, geometric shapes, or performed a number line task (47). In adults, however, while there is broad overlap in brain activations to statements on geometry, algebra, trigonometry, or complex numbers (22), the anterior LOC responded selectively to geometric sentences. This region has since been shown to be implicated in other visuospatial mathematical tasks, such as graph interpretation (72) and geometric shape processing (60). Here, our study extends these observations to children, with the left aLOC showing greater activation for geometrical sentences as opposed to arithmetic ones (Fig. 3). Moreover, we showed that the left aLOC’s selectivity for geometry remained stable across development, with no indication that the right aLOC would develop similar geometry-specific responses over time. Another study, however, showed greater activation in the right aLOC in adults than in 6-y-olds during the actual perception of geometric shapes (60) and a positive correlation between right aLOC activity evoked by visual graphics and math education (73). Together, these results suggest that the left aLOC may show a greater involvement in acquiring geometry-related words and sentences, while the right aLOC would accumulate knowledge of visual shapes and graphics—a hypothesis that should be tested in future work.

Our results also shed light on the complex issue of the developmental dynamics of prefrontal engagement in mathematics. While some studies report a developmental increase in prefrontal activation (35, 37, 51, 52), others argue that mathematical cognition progressively shifts away from frontal regions toward greater parietal reliance (27, 30–33, 36, 47). By tracking the same children over 2 y, we observed both an age-related increase in left IFG activation (Fig. 4; alongside bilateral IPS increases), but also a decrease in bilateral prefrontal activation for sentences that became better understood from T1 to T3 (Fig. 5; while IPS activity remained stable). Rather than being contradictory, these effects reflect distinct phases of a dynamic developmental trajectory. Prior studies documenting a decline in frontal activity with age have primarily relied on cross-sectional comparisons between children and adults, thus capturing only the endpoints of the developmental continuum. In contrast, studies reporting an age-related increase in frontal engagement, like the present one, have typically compared children across different ages, offering a finer-grained perspective on the developmental dynamics. Overall, the data are compatible with a quadratic model in which frontal engagement initially increases during the acquisition of formal mathematical abilities, then gradually declines as mathematical processing becomes automatized (52). Similar trajectories have been observed in the hippocampus during arithmetic processing (54), and in parieto-frontal networks during reading acquisition (63), suggesting a broader principle whereby an initial engagement of the prefrontal cortex facilitates the acquisition of complex skills before neural reorganization optimizes processing efficiency and activation shifts toward more specialized regions as expertise emerges. It should also be noted that IFG activity has been thought to reflect other processes such as metacognitive monitoring (74), which may also be expected to increase with age. However, although our data do not allow us to directly disentangle these processes, the IFG region observed in the present study appears more posterior than regions typically reported in association with metacognitive monitoring.

Since the bilateral IPS was the only region showing both an age-related increase in math activation (Fig. 4) and no significant decrease for well-mastered sentences (Fig. 5), our findings confirm its central role at the core of mathematical cognition (1, 4, 15, 16, 25–27, 49, 75) (Fig. 7). We found no evidence of hemispheric asymmetry, consistent with recent findings (35, 43, 45). Whereas prior evidence has highlighted the left IPS as a key site for acquiring symbolic mathematics (30–32, 37–41), evidence for a parallel development in the right IPS has been less consistent (33, 36). By investigating semantic processing of mathematical sentences, our study suggests that even though the right IPS is already engaged in numerical cognition from infancy (1, 26), it continues to develop alongside the left IPS during the acquisition of formal mathematical reasoning.

Although we used age as a primary predictor of brain changes, the intercorrelated variables of school exposure and mathematical ability also predicted an increased math-specific activity in several key math-selective regions, including the left ITG, left and right IPS, and left IFGop. These results mirror previous studies reporting modulations of math-induced brain activity as a function of math ability in children (37, 39, 43, 45, 76). Using model comparisons, we attempted to disentangle these developmental components and showed that mathematical ability, as measured by a numeracy screener before each fMRI session, was the strongest predictor in the left ITG and IFGop, whereas in the IPS, no single predictor clearly dominated. Given the strong intercorrelations among these variables in the present data, isolating their distinct effects remains a challenge and interpretation must remain cautious. A recent preprint took advantage of the discontinuous cutoff in school entry to scan children in whom age and school exposure were quasi-experimentally dissociated, and found that schooling was a stronger predictor of fMRI activation changes than chronological age in the parietal cortex (77). More studies of this kind are required in order to dissociate the effects of age, schooling, and ability, as was done in order to study reading acquisition (21, 78) and the math gender gap (20). Importantly, although we scanned children before and after school entry, it would be wrong to assume that learning begins at school onset. The selective activation of the math-responsive network that was observed in the youngest children, at the end of preschool, may already reflect a combination of early learning experiences (e.g., in preschool or the home environment) along with preexisting neural predispositions.

Our data allowed us to evaluate competing theories of math development and provide a more nuanced view of how the math-responsive brain network evolves during early schooling (Fig. 1). Across the first 2 y of schooling, we observed all three forms of developmental change illustrated in Fig. 1*B*, yet in partially distinct regions: an age-related amplification of activation in bilateral IPS, left IFGop, and right ITG (Fig. 4*B*); a modest recruitment of additional voxels in largely the same regions (Fig. 4*B*); and a reduced activation for well-mastered facts in bilateral ITG, left IFGop, right MFG but notably not in IPS (Fig. 5*B*). These mechanisms are thus not mutually exclusive but characterize complementary aspects of developmental change. The recruitment of new cortical territory for mathematics raises important questions for future research. Did these voxels exhibit another functional selectivity before becoming tuned to mathematical content? Were they initially unresponsive, or were selectively engaged in other functions, only to be later repurposed for math processing, in line with the neuronal recycling hypothesis (21)? This question remains largely unexplored in the field of mathematical cognition, in contrast to reading research (63, 79, 80). One plausible hypothesis is that IPS sites initially involved in visuo-spatial operations are repurposed for encoding the abstract numerical or geometric content of words and sentences—a possibility supported by the known overlap of these representations in the IPS of both children and adults (47, 71, 81, 82).

Finally, our sentence-based design allowed us to probe developmental change in neural space (Fig. 1*C*). Inspired by advances in the theoretical and empirical description of coding by high-dimensional neural subspaces (64–67), we estimated the ID of the neural space occupied by the same mathematical sentences over time. We found a small yet significant increase in dimensionality in math-related regions (Fig. 6). This finding suggests that, as children mature, the complexity of the neural manifold that encodes mathematical contents increases. This finding offers a potential solution to the vexing question of how new mathematical facts, including advanced ones, can be acquired within the same overall cortical circuit: new concepts can be encoded using previously unused directions of neural vector space. This interpretation remains tentative, however, as the increase in dimensionality did not reach significance within specific math-related ROIs after FDR correction. Replication with higher signal-to-noise methods will therefore be essential, using either ultra-high-field imaging or human intracranial recordings, which have recently been shown to capture various semantic dimensions of words and sentences (83, 84), including numbers (85).

To our knowledge, no previous study has examined how spatial neural complexity evolves during development—except for one, which used representational similarity analysis and found less differentiated neural representations in children with math learning difficulties compared to typically developing peers (76). Temporal complexity has been more extensively explored, especially through entropy measures in electrophysiology (86–88) and fMRI (50, 89). These studies generally report increasing temporal complexity from childhood to adulthood, suggesting that the time domain may offer an additional axis for coding flexibility during development.

The present methods, involving longitudinal recordings of brain activity patterns evoked by the same sentences over development, offer a tool for conceptualizing and measuring the impact of education on the brain. While we focused here on mathematical development, this dataset also holds rich potential for exploring developmental changes in social, linguistic, and musical cognition.

## Materials and Methods

### Participants and Task.

#### Ethics.

All experiments were approved on December 21, 2022, by the French national ethical committee (CPP: *Comité de Protection des Personnes,* CPPIDF-2022-MS276). Written informed consent was obtained from both parents or legal guardians prior to participation, and written assent was obtained from the children before each testing session. Families were reminded that participation was voluntary and that they could withdraw at any time.

#### Participants.

58 children were recruited in spring 2022 and participated in an initial MRI session (T1) during summer-fall 2022. At the time, children were 5 to 6 y old, either finishing kindergarten or entering first grade. Five were excluded after T1: one did not wish to continue, one had attention disorders, and three had language disorders (exclusion criteria). 53 participants (27 boys, 26 girls, mean age = 6.38 ± 0.35 y, ages range = [5.67, 6.99]) were therefore included in T1 analysis. 1 y later (May to October 2023), 46 of these participants (24 boys, 22 girls, mean age = 7.16 ± 0.30 y, ages range = [6.57, 7.60]) returned for a second session (T2). Finally, 35 of them (16 boys, 19 girls, mean age = 8.07 ± 0.33 y, ages range = [7.47, 8.70]) returned for a third session (T3), from May to October 2024, corresponding to the end of second grade or start of third grade. Participants were recruited from schools in the priority education network in the north of Île-de-France (serving low-income families). On average, mothers had 0.92 y of education beyond high school, with 25 on 53 having a *baccalauréat* (BAC) or less, while fathers had 0.28 y beyond high school, with 31 on 53 having a BAC or less. Sample sizes were not predetermined using statistical methods, but were comparable to or larger than those used in recent cross-sectional or longitudinal studies (35, 38, 47, 63, 80).

#### Stimuli.

72 sentences (24 in each cognitive domain) and 24 melodies were created. The sentences covered three cognitive domains: mathematics (e.g., “Three plus three is six”; hereafter called Math), general nonmathematical knowledge about biology (e.g., “The ant is a black insect”; called GK), and social sentences (e.g., “According to the policeman, thieves are nice,” called Social). Within each domain, 12 statements were true and 12 were semantically false. All statements were recorded by a female native French speaker and were matched for syntactic structure, number of words (in average 7.5 words per sentence in the math, GK, and social conditions), number of phonemes (in average 19.9, 20.2, and 22.6 phonemes per sentence for respectively math, GK, and social), duration (in average 3.46 s, 3.52 s, and 3.46 s for respectively Maths, GK, Social), and mean word frequency in French. A detailed list of statements is presented in *SI Appendix*, Fig. S1*A*. The melodies were equalized in duration and intensity, and half of the melodies were consonant. In dissonant melodies, one of the final two notes was shifted one tone away from the expected key. The musical condition is not analyzed in the present article.

#### Paradigm.

Children were presented with the sentences and musical melodies in random order and asked to judge whether the sentences were true or false, and the melodies normal or weird. Each trial began with a beep, followed by the presentation of a sentence or melody. At the end of the presentation, participants had 3 s to press a right button for correct/normal, or a left button for incorrect/weird. Visual and auditory feedback were provided at the end of the 3 s, followed by an intertrial interval varying between 1s and 3 s (average = 2 s). The whole experiment included 4 runs of 24 pseudorandomized trials (eight sentences/melodies, with four correct and four incorrect, from each condition), with each run lasting approximately 3′30″. However, as children could opt out, they almost never completed the entire experiment: 1.54 ± 0.30 runs/child at T1; 1.85 ± 0.33 at T2; and 2.29 ± 0.40 at T3.

#### Data quality and inclusion criteria.

A total of 30% of T1 runs, 46% of T2 runs, and 27% of T3 runs were excluded from the analysis due to excessive motion (i.e., >3 mm). The final sample included 40 children in T1, 24 in T2, and 30 in T3, each with at least one acceptable run, for a total of 94 time points. Among the included children, the overall amount of motion and the average tSNR did not significantly differ across periods; nor were significantly correlated with children’s age or with the number of days the child spent at school (*SI Appendix*, Fig. S2 and *SI Appendix*).

#### Experimental procedure.

Before each fMRI session, to enhance the quality of data, children were trained in a mock scanner. They listened to a recording of the noises associated with all fMRI sequences and were trained on the fMRI task with four example trials. They received live feedback on their motion. Following this ~20 min practice session, children proceeded to the actual MR scanner. Each session began with the acquisition of anatomical images (see below), followed by one functional run of a visual categories localizer task (not analyzed here), one run of the sentence comprehension task, a diffusion tensor imaging sequence (not analyzed here), and then alternating runs of visual localizer and sentence comprehension tasks until the child opted to end the session. To minimize head motion, the quality of each sequence and functional run was reviewed immediately after acquisition, and children received verbal feedback accordingly.

#### School exposure.

School exposure was estimated by the number of days children had spent at school at the time of scanning. Since we did not have access to data on school attendance, this number was approximated by computing the number of school days elapsed between the beginning of first grade and the date of the fMRI scan, according to the official school calendar.

### Behavioral Experiments.

On the same day as the MRI session, we performed behavioral tests and experiments with the children, to 1) verify that they had no learning disabilities (exclusion criteria), and 2) quantify their individual abilities in different domains (e.g., motricity and oral comprehension). Here, we analyzed two of them, which we describe in the next sections.

#### Numeracy screener.

Each child’s mathematical ability was assessed by a behavioral test, the numeracy screener (90), conducted on the same day as each of their fMRI session. This paper-and-pencil test evaluates children’s ability to compare both symbolic and nonsymbolic numerical magnitudes. During each trial, children were instructed to cross out the larger of two single-digit numerical magnitudes. The test comprised two conditions: a symbolic condition, in which children compared 56 pairs of Arabic numerals, and a nonsymbolic condition, where they compared 56 pairs of dot arrays. Each condition lasted 2 min, with the order of conditions randomized across participants. For each condition, the number of errors was recorded, along with the time taken to complete the task (if completed within the 2-min limit). An overall mathematical ability was then computed for each child, at each time point, as

$$Math\;Ability=\frac{Accuracy(Symbolic)}{2*Time(Symbolic)}+\frac{Accuracy(Non-Symbolic)}{2*Time(Non-Symbolic)}.$$

#### Reading fluency.

Reading fluency was quantified as the mean number of correctly read words during a 1-min timed reading task (91).

### fMRI Parameters and Analysis.

#### MRI data acquisition.

MRI acquisition was performed on a 3-Tesla scanner (Siemens Prisma) equipped with a 64-channel head-coil. T1-weighted structural images were acquired [repetition time (RT) = 2300ms; echo time (TE) = 2.98 ms; voxel-size = 1 mm^3^]. Functional images were obtained using a high-resolution multiband imaging sequence (RT = 1,810 ms; TE= 30.4 ms; voxel-size = 2 mm^3^; multiband factor = 3). Each run comprised 142 functional scans covering the whole brain (69 slices). To estimate distortions, two spin‐echo field maps with opposite phase encoding directions were acquired: one volume in the anterior‐to‐posterior direction (AP) and one volume in the other direction (PA). Children were wearing noise-protection earphones.

#### MRI preprocessing.

Preprocessing was performed with the standard pipeline fMRIprep (version 22.0.2). A detailed description of the preprocessing steps generated by fMRIprep is provided in *SI Appendix*.

#### fMRI GLM models.

fMRI first-level models were estimated for each subject to model task-related blood-oxygen-level dependent (BOLD) responses. The design matrix included four experimental conditions: math, GK, social, and music. Two regressors were included to account for button responses (left, right). Confound regressions included six motion corrections to account for head translation and rotation along the three axes, as well as three regressors for physiological noise: average signal across all brain voxels, average CSF signal, and average WM signal. To account for low-frequency signal drifts, polynomial drift models from constant to 5th order were added. Finally, a constant term was also included to model the baseline signal. Group-level analysis was performed using a second-level model with spatial smoothing applied at a full-width half-maximum (FWMHM) of 8mm. Unless indicated, all brain activation results are reported using a voxel-wise threshold of *P* < 0.001, corrected for multiple comparisons across the whole brain using false discovery rate (FDR) at α < 0.05. We performed two additional control analyses to ensure that the math-responsive network was independent of sentence complexity: i) we repeated the first-level analysis using only trials that were correctly judged by children (*SI Appendix*, Fig. S3*A*), and ii) we added a trial-by-trial RT-difficulty regressor, computed as each trial’s RT minus the participant’s mean RT, to the first-level model (*SI Appendix*, Fig. S3*B*).

#### Regions-of-interests (ROIs) derived from adult fMRI data.

To assess whether the children’ neural networks resemble those observed in adults, we used fMRI data collected in a cohort of adult participants, with a similar paradigm (56). In this paradigm, adults were asked to evaluate the truthfulness of sentences belonging to different semantic conditions (e.g., mathematics). From the activation maps obtained for the contrast math vs. nonmath sentences at the group-level, we defined eight math-responsive ROIs: left and right intraparietal sulcus (IPS), left and right inferior temporal gyrus (*ITG*), left and right inferior frontal gyrus pars opercularis (IFGop) and left and right medial frontal gyrus (MFG) (*SI Appendix*, Fig. S4*A*). We note that the frontal ROI labeled as IFGop in adults corresponds to a relatively large cluster that may extend dorsally into adjacent superior frontal regions (SFG). Similarly, from the activation maps obtained for the contrast social vs. contextual knowledge sentences (i.e., referring to a person vs. facts in a specific context: “For Einstein the atomic bomb is a harmless object” vs. “In the Amazon spiders are harmless animals”), we defined seven social-responsive ROIs: bilateral temporal gyrus (TP), left angular gyrus (L AG), right temporo–parietal junction (R TPJ), left medial frontal gyrus (L MFG), medial frontal gyrus (MedFG), and precuneus (*SI Appendix*, Fig. S4*B*). Finally, from the activation maps obtained for geometry vs. other mathematical sentences (e.g., “A rectangle has only one axis of symmetry” vs. “Three multiplied by twelve is thirty-six”), we defined two *geometrical-responsive* ROIs: the left and right aLOC (*SI Appendix*, Fig. S4*C*). Within each region-of-interest, we identified the 10% of voxels with highest activation peak for adults for the contrast in question (e.g., math vs. nonmath). The β-values extracted in children in each region were computed from these 10%-voxels.

Children’s fMRI data were normalized to the MNI152NLin2009cAsym template, while the adult data were normalized to the Montreal Neurological Institute avg152 template. To extract the β-values from the correct voxels, we transformed the children’s statistic maps into the adult template first, before applying spatial filtering.

To address concerns about ROI definition, we additionally defined child-derived ROIs. These were computed from the group-level activation map computed across all children, regardless of age, for the math vs. nonmath contrast (Fig. 2*A*). From this map, we defined nine child math-responsive ROIs: left and right IPS, left and right ITG, left and right *IFGop* and left and right MFG, and left SFG. These child-derived ROIs were used in a supplementary analysis to test the robustness of age-related effects to ROI definition (*SI Appendix*, Fig. S8).

#### Data analysis.

Details of the statistical analyses are provided in *SI Appendix*, including descriptions of a) mixed-model regressions, b) longitudinal analyses, c) model comparisons, and d) ID computations.

## Supplementary Material

Appendix 01 (PDF)

## Data Availability

All code used for data preprocessing, statistical analyses, and figure generation is available on the Open Science Framework (OSF) at https://osf.io/5kvd9/overview?view_only=52f336a5f13f446eb569ada6c798cf31 (92). All other data are included in the manuscript and/or *SI Appendix*.
