# Supplementary material for: Initial organization and progressive expansion of the math-responsive brain network during the first school years
Source: Proc Natl Acad Sci U S A. 2026 Jul 2;123(27):e2602515123. doi: 10.1073/pnas.2602515123 (PMC13342980; doi:10.1073/pnas.2602515123)
Supplement: Supplementary file 1 — Appendix 01 (PDF) [file pnas.2602515123.sapp.pdf]

PNAS

Supporting Information for

Initial organization and progressive expansion of the math-responsive brain network during the first school years

Théo Morfoisse[1,2*], Severine Becuwe[2], Marie Palu[2], Cassandra Potier-Watkins[1], Ghislaine Dehaene-Lambertz[2], Stanislas Dehaene[1,2]

Corresponding author: Théo Morfoisse and Stanislas Dehaene

Emails: theo.morfoisse@gmail.com and stanislas.dehaene@college-de-france.fr

This PDF file includes:

Supporting text
Figures S1 to S11
Tables S1 to S12

Supporting Information Text

Results

Age-related increases in mathematical selectivity. Linear mixed-model regression revealed significant age-related increases in math selectivity in three of the math-related ROIs: both IPS and the left IFGop. To ensure that these age-related increases in $\Delta\beta$-values were not driven by age-related differences in data quality, we conducted two additional linear mixed-model regressions within each ROI, including either a head motion or temporal signal-to-noise (tSNR), as covariates. Head motion was not significant in any ROI (all $p > .12$) and age remained a significant predictor in the same regions with nearly unchanged p-values (table S5). Although tSNR emerged as a predictor in two regions – the right ITG ($t(83.49) = 2.85$, $p = .006$, $p_{FDR} = .051$) and the left MFG ($t(90) = 2.19$, $p = .031$, $p_{FDR} = .099$) - age effects persisted in all initially identified regions, again with nearly unchanged p-values (table S6). These control analyses confirmed that $\Delta\beta$-values changes reflect genuine development effects rather than variations in measurement quality over time.

Methods

Data quality and inclusion criteria. Among the included children, the overall amount of motion (averaged across axes and runs) did not significantly differ across periods (T1 vs. T2: $t(62.1) = -1.71$, $p = .21$; T1 vs. T3: $t(57.4) <1$; T2 vs. T3: $t(60.3) <1$; obtained from linear mixed-model regressions, with participant as random effect), nor were significantly correlated with children's age ($t(66.14) <1$), or with the number of days the child spent at school ($t(59.55) <1$) (figure S2A). Despite this absence of differences, motion parameters were included as regressors in the fMRI data analysis. Motion curves for each subject and each run are reported in the supplementary data. Similarly, the average temporal signal-to-noise (tSNR) – i.e., the mean of the tSNR across voxels - computed for each included scanning did not significantly differ across periods (T1 vs. T2: $t(59.7) = 1.41$, $p = .34$; T1 vs. T3: $t(55.6) <1$; T2 vs. T3: $t(57.8) = -1.42$, $p = .34$) and was not significantly correlated with children's age ($t(61.33) <1$) or with the number of days the child spent at school ($t(55.34) <1$) (figure S2B).

MRI preprocessing. The following description was generated by fMRIprep.

Anatomical Data: The T1-weighted (T1w) images was corrected for intensity-non-uniformity (INU) and used as T1w-reference throughout the workflow. The T1w-reference was then skull-stripped using a target template (OASIS30ANTs). Brain tissue segmentation of cerebrospinal fluid (CSF), white-matter (WM) and gray matter (GM) was performed on the brain extracted T1w. Volume-based spatial normalization to two standard spaces (MNI152NLin6Asym, MNI152NLin2009-cAsym) was performed through nonlinear registration using brain-extracted versions of both T1w reference and the T1w template.

Functional data preprocessing: First, a reference volume and its skull-stripped version were generated using a custom methodology of fMRIPrep. Head-motion parameters with respect to the BOLD reference (transformation matrices, and six corresponding rotation and translation parameters) were estimated before any spatiotemporal filtering. BOLD runs were slice-time corrected. The BOLD time-series (including slice-timing correction) were resampled onto their original, native space by applying the transforms to correct the head-motion. The BOLD reference was then coregistered to the T1w reference. Co-registration was configured with six degrees of freedom. Several confounding time-series were calculated: framewise displacement (FD), DVARS (Derivative Variance Across Space) and three region-wise global signals. FD and DVARS are calculated for each functional run. The three global signals are extracted within the CSF, the WM, and the whole-brain masks. These steps were performed for each of the BOLD runs.

Data Analysis

Mixed-model regressions. We employed mixed-model regression to account for the hierarchical structure of the data (repeated measures within each participant), and because the number of observations and participants' ages varied. Mixed-model regressions were used both to 1) characterize the math-responsive network, by examining differences in activations between different conditions (e.g., math vs. social), and 2) investigate the development of this math-responsive network, by studying the evolution of these activations with age.

Degrees of freedom for fixed effects in the linear mixed models were approximated using Satterthwaite's method, which accounts for both the hierarchical structure of the data and the estimation uncertainty of random effects. These degrees of freedom were then used to estimate p-values for the fixed effects. False Discovery Rate (FDR) correction was applied to control for multiple comparisons using the Benjamini-Hochberg procedure.

All regressions performed are described just below.

To examine differences in performance during development, we conducted a binomial mixed-model regression with trial-by-trial performance as dependent variable, condition (math, general knowledge, social) and age as fixed effects, and participant as a random effect (table S1). Similarly, to examine differences in reaction times during development, we performed a linear mixed-model regression with reaction times in correct trials as dependent variable, condition (math, general knowledge, social) and age as fixed effects, and participants as a random effect (figure S1B, table S2).

To compare brain activations within each of the math-selective ROI, we performed a linear mixed-model regression, with bold signal (%) at each time step as dependent variable, condition (math, general knowledge, social) as fixed effect and participant as a random effect (figure 2B). Similar analyses were conducted separately in each of the periods (T1, T2, and T3; figure S6).

To compare brain activations within each of the geometrical-selective ROI, we performed linear mixed-model regression, with β-values as dependent variable, condition (geometry, arithmetic, general knowledge and social) as fixed effect, and participant as a random effect (figure 3B). We conducted similar frequentist linear mixed-model regression, as well as Bayesian ones in the math-selective ROI, comparing only the geometrical and arithmetical β-values (figure 3B, table S3). To investigate the brain activations along the y-axis with both left and right IPS, we performed mixed-model regressions with $\Delta\beta$-values ($\beta_{Arithmetic} - \beta_{Geometry}$) as the dependent variable, y-axis as the fixed effect and participant as a random effect (figure S7B).

To track the development of math-responsive brain network, we conducted, within each of the math-responsive ROI, a linear mixed-model regression with $\Delta\beta$-values ($\beta_{Math} - \beta_{Non-Math}$) as dependent variable, age as fixed effect and participant as a random effect. To capture potential non-linear effects, the model included a second-order polynomial term for age (figure 4A, table S4). Given that the quadratic term was not significant in any of the regions tested, only a linear term was included in subsequent analyses. To evaluate hemispheric differences, we performed similar linear mixed-model regressions with $\Delta\beta$-values ($\beta_{Math} - \beta_{Non-Math}$) as dependent variable, age and hemisphere as fixed effects and participant as a random effect, within each set of bilateral math-responsive ROI (e.g., IPS).

Following the approach in Nord et al. (2021), to ensure that these age-related increases in $\Delta\beta$-values were not driven by age-related differences in data quality, we conducted two additional linear mixed-model regressions within each math-selective ROI, including either age and average head motion during, or age and averaged temporal signal-to-noise as fixed effects, $\Delta\beta$-values ($\beta_{Math} - \beta_{Non-Math}$) as dependent variable, and participant as random effect (table S5, S6).

To examine other developmental factors, we also conducted linear mixed-model regressions within each math-responsive ROI, with the same dependent variable as above (i.e. $\Delta\beta$-values ($\beta_{Math} - \beta_{Non-Math}$), participant as a random effect, and math abilities as fixed effect (table S7).

Finally to assess how neural dimensionality evolves with age, we performed, within each set of ROIs (either math-responsive ROIs, or social responsive ROIs) and for each condition (math, general knowledge, and social), linear mixed-model regressions, with dimensionality-values within each ROI as dependent variable, age as fixed effect, and included random intercepts and slopes for age per participant and a random intercept for ROI, to take into account that each participant contributed multiple measurements across brain regions and age points (figures 6B and S10A). We also performed similar regressions, in each of the set of ROIS, with same dependent variables, random intercepts and slopes, but with age and condition as fixed effect. We also performed simple linear regression in each of the math-responsive ROI, without any random effect due to insufficient variance across participants (figure S11B, table S12).

Longitudinal analyses. In two analyses, we took advantage of the longitudinal design of our dataset by focusing on participants who completed T1 and T3 sessions (26 children in total). Instead of using mixed-model regressions, we directly examined changes in activation patterns either at the voxel level or for individual sentences. The first analysis tracked voxel-level activation changes within individuals over the two-year interval (figure 4B). Within each math-responsive ROI, voxels were categorized into five categories based on their math-selective activation ($\Delta\beta = \beta_{Math} - \beta_{Non-Math}$) at T1 and T3: *never activated voxels* ($\Delta\beta(T_3) < 0, and\ \Delta\beta(T_1) < 0$), *dropout voxels* ($\Delta\beta(T_3) < 0, and\ \Delta\beta(T_1) > 0$), *recruited voxels* ($\Delta\beta(T_3) > 0, and\ \Delta\beta(T_1) < 0$) and finally, within the voxels activated in both periods ($\Delta\beta(T_1) > 0$ and $\Delta\beta(T_3) > 0$), those whose activity was *reduced* ($\Delta\beta(T_3) < \Delta\beta(T_1)$) or *amplified* ($\Delta\beta(T_3) > \Delta\beta(T_1)$). In each participant, we computed the percentage of voxels in each of these five conditions, and we computed simple linear regressions to compare the percentages of *amplified* and *reduced* voxels (table S8), and the percentages of *recruited* and *dropout* voxels (table S9).

The second analysis assessed how brain responses to the same sentences evolved across time in the same children. Each of these 26 children heard an average of 10 $\pm$ 2.65 sentences repeated across the two sessions. For each sentence, we computed the average behavioral performance across children at both T1 and T3 and used the difference ($\Delta Perf$) as an index of comprehension improvement. For each sentence and each child, we computed the change in brain activation ($\Delta\beta$-values) by subtracting the β-values at T3 from those at T1, within each set of ROIs (either math-responsive ROIs or social-responsive ROIs). We then performed simple linear regressions between $\Delta\beta$-values, averaged across children and set of ROIs, and $\Delta Perf$, within math-responsive ROIs (figure 5A) and within social-responsive ROIs (figure S10A). We further examined whether this relationship differed significantly across conditions, by including condition as an additional predictor in the model ($\Delta\beta$-values $\sim \Delta Perf \times Condition$) and examining the interaction term. We also conducted similar analysis within each math-responsive ROI (figure 5B, table S10) and within each social-responsive ROI (figure S10B, table S11), with $\Delta\beta$-values averaged across children.

We chose to compare T1 and T3, rather than T1–T2 or T2–T3, for two main reasons: (1) these timepoints represent the two endpoints of the longitudinal span, offering maximal contrast, and (2) the sample size of included children was smallest at T2.

Comparison of models. We assessed model fit using the Akaike information criterion (AIC), which balances goodness of fit against model complexity (i.e., the number of predictors). The model with the lowest AIC value was considered the best-fitting model. To evaluate the robustness of model differences, we computed the Akaike weights, which estimates the probability that a given model is the best model, considering the evidence from all other models. For each model, the Akaike weight was computed as:

$$w_i = \frac{\exp\,(-0.5 \cdot \Delta AIC_i)}{\sum_{j=1}^{K} \exp\,(-0.5 \cdot \Delta AIC_j)}$$

were $\Delta AIC_i = AIC_i - \min\,(AIC)$.

Intrinsic dimensionality. For each run performed by each child, and for each condition (Math, general knowledge, Social) within each region-of-interest or set of region-of-interest, we performed a Principal Component Analysis (PCA; figure 6A). Specifically, we conducted the neural covariance matrix based on a data matrix **B** of shape $6 \times N_{voxels}$, where each row corresponds to one of the six sentences presented during the run, and each column represents a voxel's activation. When performed within each region-of-interest, N is the number of voxels within this region. When performed within a set of regions, N is the number of voxels within all mathematical or social regions. We then extracted the eigenvalues (λ_1, λ_2, …, λ_6) of this covariance matrix, which indicate the variance explained by each principal component. To estimate the intrinsic dimensionality of the neural activation patterns, we used the "effective dimensionality" or "participation ratio", defined as

$$D = \frac{\left(\sum_{i=1}^{M} \lambda_i\right)^2}{\sum_{i=1}^{M} {\lambda_i}^2}$$

This measure captures how many dimensions are effectively needed to represent most of the data. It reaches its minimum value (1) when all variance is concentrated in a single component (e.g., $\lambda_1 = 1$, $\lambda_{i>1} = 0$), and its maximum value (M) when all components contribute equally (i.e., $\lambda_i = \lambda_k, \forall i$).

Supplementary Figures

A. Stimuli

Math		General Knowledge		Social	
Sentence	Accuracy (%)	Sentence	Accuracy (%)	Sentence	Accuracy (%)
Le nombre sept est plus grand que cinq	84±5	La fourmi est un insecte noir	71±6	Le père noël adore les petits enfants	90±4
Quatre est le double de deux	46±6	Les pommes poussent dans les arbres	81±5	Les jardiniers adorent les arbres	76±7
Un rectangle possède quatre côtés	59±6	Les chevaux mangent de l'herbe	83±5	Les enfants ont peur des monstres	73±7
Un triangle doit avoir trois côtés	71±8	Les pommes peuvent être rouges	84±5	Le garagiste veut réparer les voitures	83±5
Le nombre trois est plus grand que six	80±5	Les clémentines sont des légumes verts	82±5	Les sorcières détestent les citrouilles	57±8
Quatre est le double de trois	50±6	Le pigeon est un oiseau chanteur orange	66±6	Les princesses ont peur des licornes	63±8
Un triangle possède quatre côtés	67±6	Les chiens sont des oiseaux marrons	76±6	Les policiers adorent les menteurs	65±7
Un cercle c'est la même chose qu'un carré	85±6	Les fourmis ont une belle fourrure	52±7	Les pompiers ont peur du feu	65±6
Deux plus trois est égal à cinq	66±5	Les arbres perdent leurs feuilles en automne	65±7	D'après les sorciers, la magie existe	50±6
Six moins deux est égal à quatre	51±6	Le lion est un animal sauvage de la savane	70±7	Pour les sorcières, les grenouilles sont délicieuses	65±7
Quatre se trouve entre deux et neuf	21±5	Le chameau est un animal à bosses	76±7	Selon l'infirmière, le vaccin est une bonne chose	83±5
Dans un carré, on peut compter quatre côtés	68±7	Les poissons peuvent respirer sous l'eau	79±5	D'après la maîtresse, les enfants doivent rester sages	92±3
Trois plus quatre est égal à cinq	69±6	Le lapin est l'animal le plus rapide du monde	70±5	Selon le petit chaperon rouge, le loup est gentil	82±5
Neuf moins un est égal à deux	60±7	La baleine bleue est un poisson de rivière	52±7	Selon la maîtresse, les enfants parlent tous le chinois	58±6
Cinq se trouve entre sept et douze	76±6	Le dauphin est un animal de compagnie	71±6	D'après le policier, les voleurs sont gentils	88±4
Un rectangle est une forme avec cinq côtés	71±8	La noix de coco est un arbre à fleurs	60±6	D'après les pompiers, le feu est une bonne chose	79±6
Si on ajoute deux et trois, cela donne cinq	65±7	Le requin est un animal qui vit dans l'eau	83±5	Les policiers pensent que les voleurs sont méchants	71±6
Quand on additionne deux et deux, cela fait quatre	90±4	La poule est un oiseau qu'on élève à la ferme	66±5	La maîtresse veut que les enfants soient sages	98±2
Quand on enlève un à trois, cela donne deux	56±7	Le chameau est un animal qui marche dans le désert	84±4	Le boucher pense que la viande est délicieuse	78±5
Quand on dessine un ballon, cela donne un cercle	76±7	Le papillon est un insecte qui a des ailes	91±5	Le père noël pense que les fêtes de noël sont joyeuses	96±3
Si on ajoute deux et un, cela donne neuf	73±6	Le chat est un animal qui a des plumes	64±7	Le boulanger pense que le pain est mauvais pour la santé	58±6
Quand on additionne un et deux, cela fait sept	78±5	Les dinosaures sont des animaux qui existent encore	78±5	Le jardinier pense que les plantes sont inutiles	62±7
Pour dessiner un livre, on fait un cercle	72±5	Le singe est un animal qui aboie fort	71±7	Les adultes croient que les dragons existent	89±4
Si on colle deux ronds, on obtient un triangle	65±5	L'abeille est un poisson qui a une queue	72±5	Le docteur pense que tous les médicaments font mal	65±7

B. Response Times

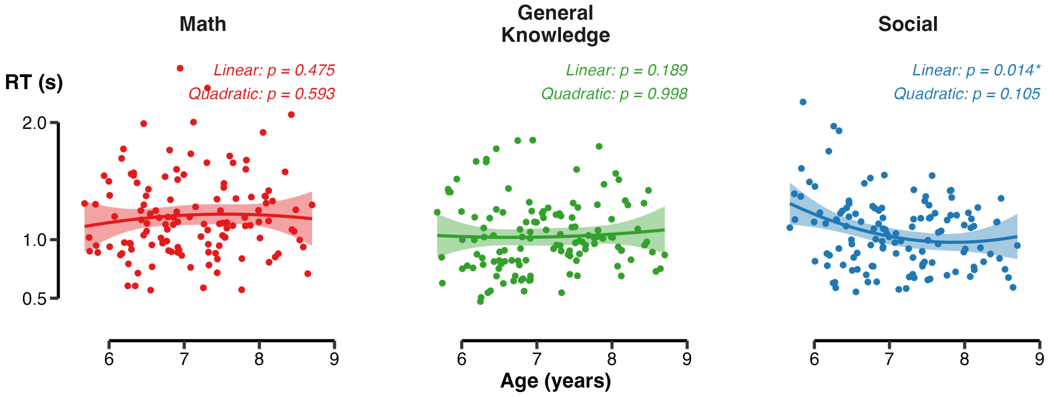

Fig. S1. (A) Stimuli. French sentences used during the fMRI task, with 24 sentences covering different mathematical concepts, 24 of general knowledge and 24 of social reasoning. Different levels of complexity were included for each semantic condition. We reported the average accuracy and the standard error across participants and period for each individual sentence. **(B)** Reaction times (s) as a function of age's participants, for correct trials only. A mixed-model regression was performed for each condition, with age as fixed effect and participant as random effect (*$^*p < 0.05$; $^{**}p < 0.01$; $^{***}p < 0.001$*).

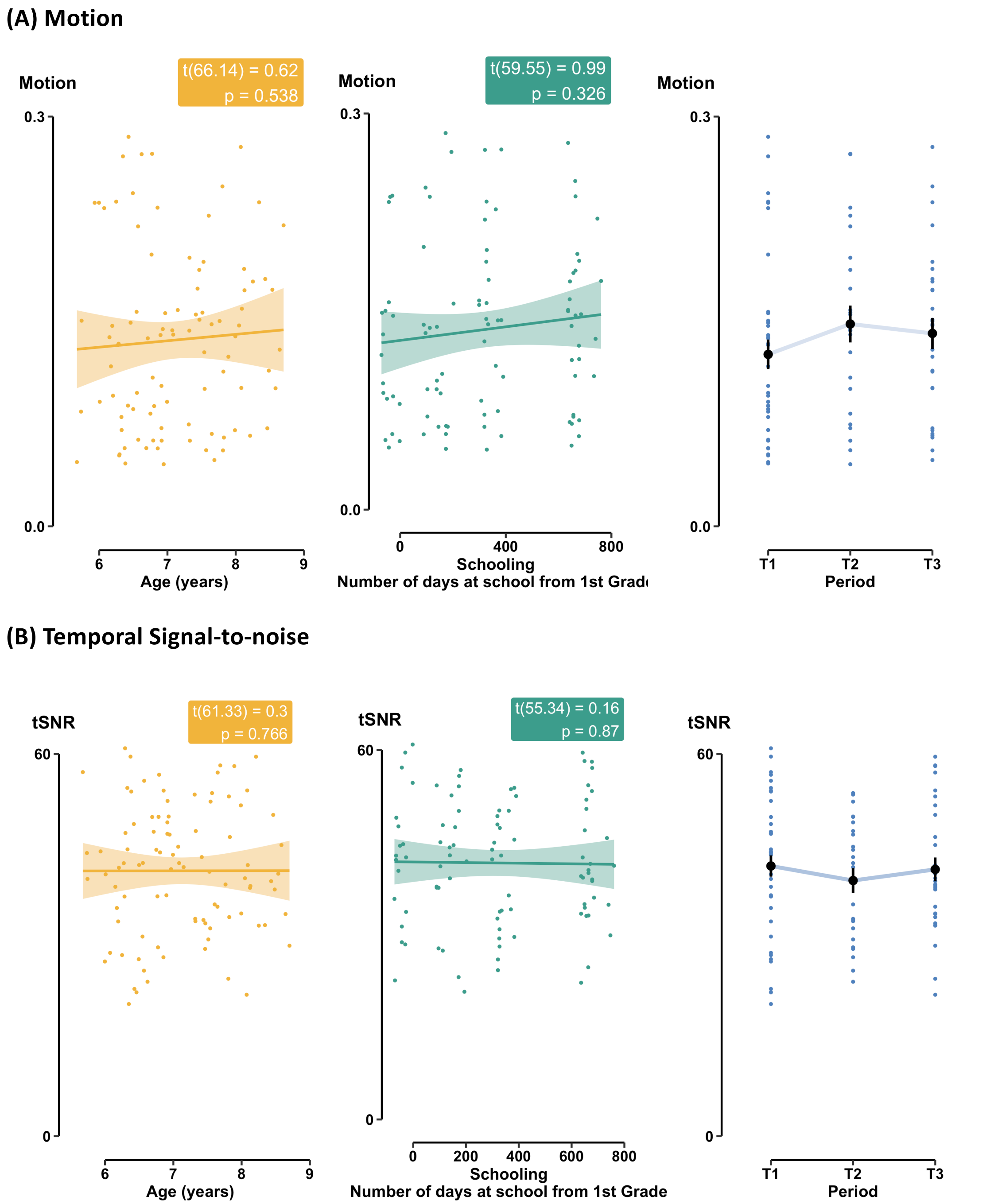

Fig. S2. Data quality. (A) Overall amount of motion was computed for each included participant, averaged across axes and runs. Mixed-model regressions were performed with either age, number of days spent at school, or period as fixed effect, and participant as random effect (*$^*p < 0.05$; $^{**}p < 0.01$; $^{***}p < 0.001$*). **(B)** Mean temporal signal-to-noise ratio (tSNR), averaged across voxels, were computed for each included participant. Mixed-model regressions were performed with either age, number of days spent at school, or period as fixed effect, and participant as random effect (*$^*p < 0.05$; $^{**}p < 0.01$; $^{***}p < 0.001$*).

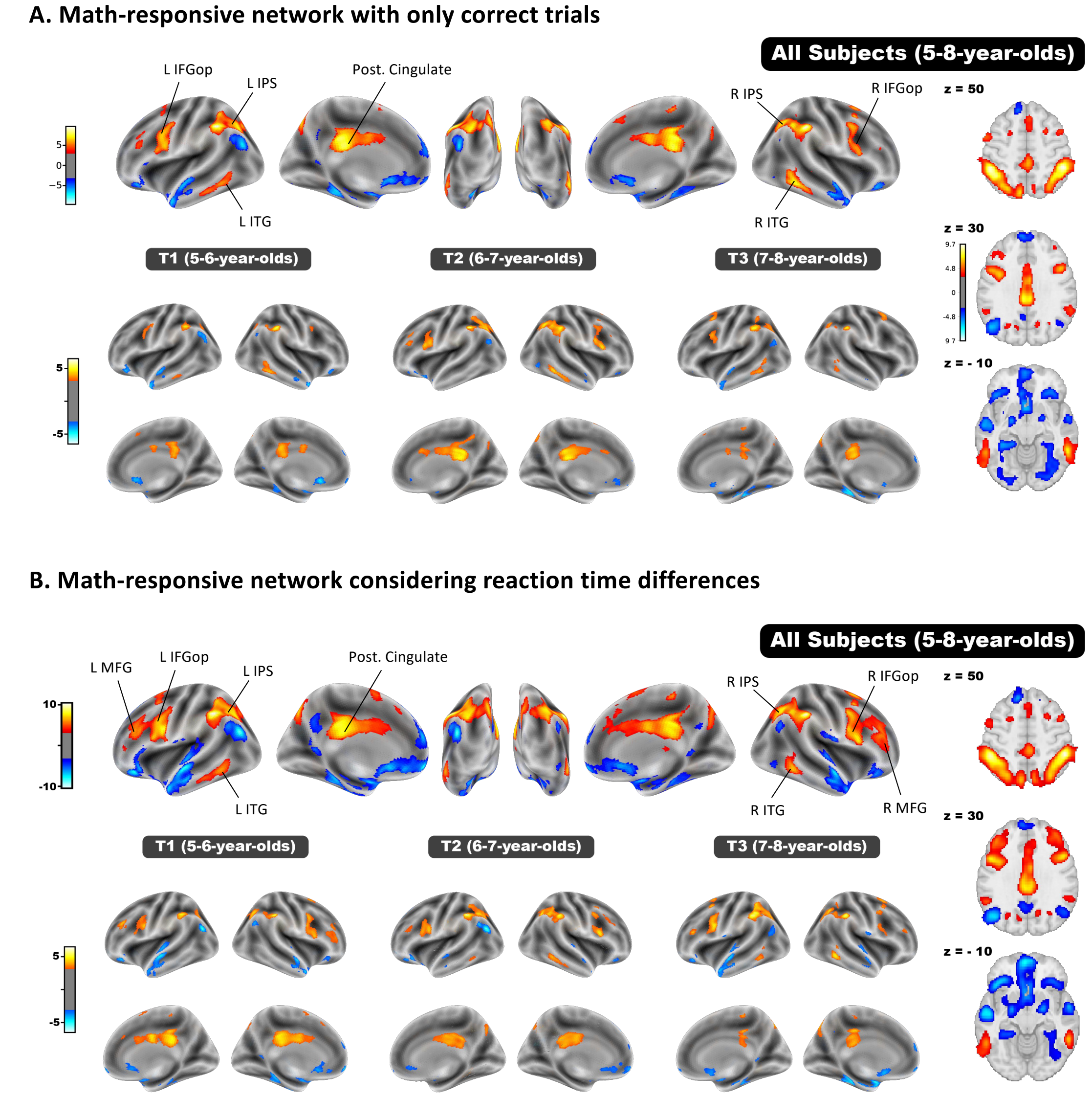

Fig. S3. Control analysis for figure 2, in the same format. **(A)** Controlling for differences in accuracy across conditions: fMRI contrast between math and non-math sentences, restricted to trials correctly judged by children. **(B)** Controlling for differences in response time across conditions: fMRI contrast between math and non-math sentences, including response time for each sentence as a first-level covariate.

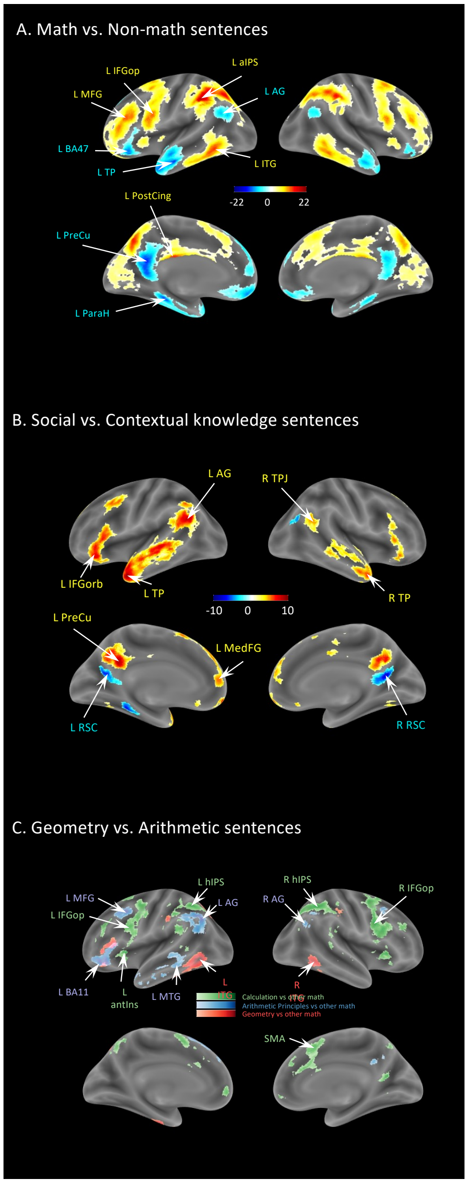

Fig. S4. Adult networks from Moreno et al. (2025). Activation maps obtained in an independent cohort of adults, who were also asked to assess the veracity of sentences belonging to different semantic conditions. The sentences were not the same as those used in children. **(A)** Activation maps obtained for the contrast math vs. non-math sentences. **(B)** Activation maps obtained for the contrast social vs. contextual knowledge sentences (i.e., referring to a person vs. facts in a specific context). **(C)** Activation maps obtained for the contrast geometry vs. other mathematical sentences.

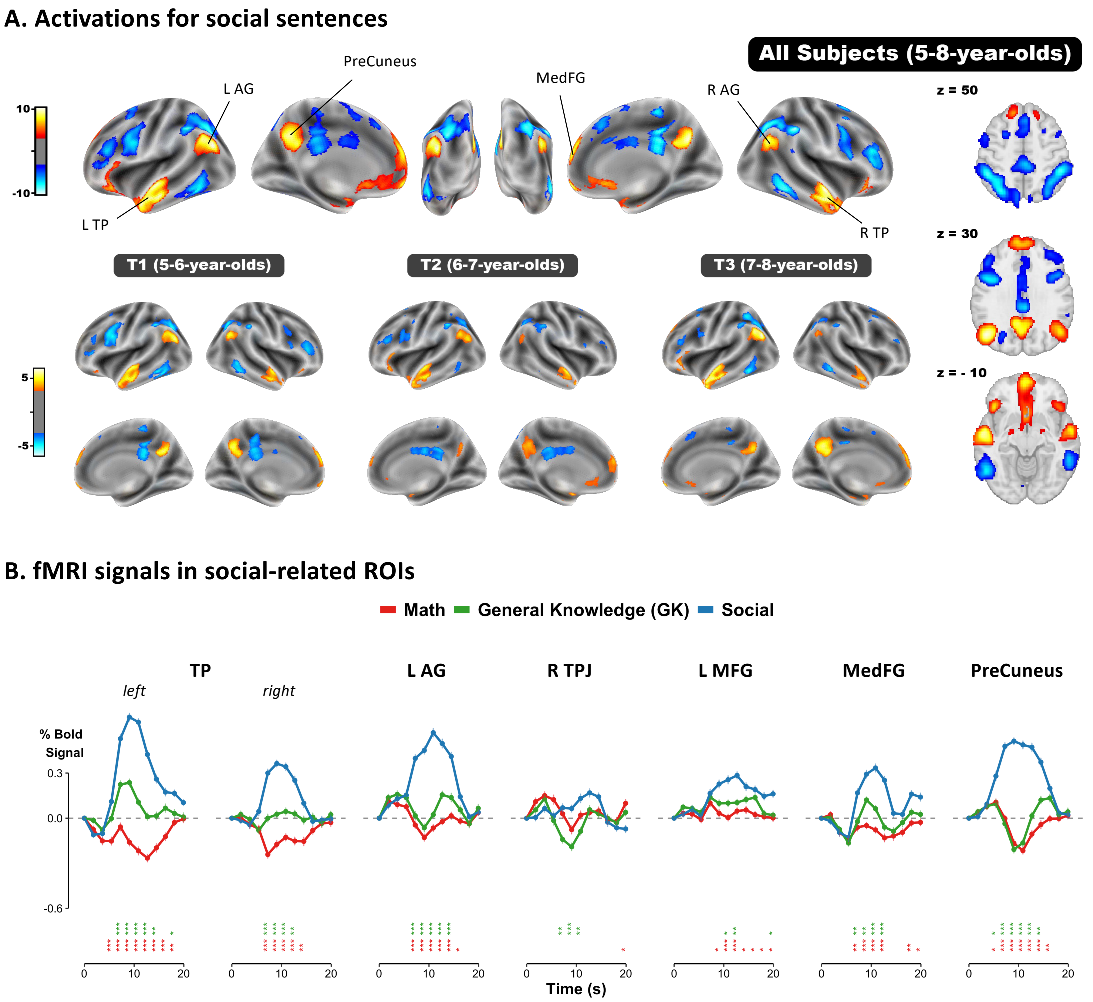

Fig. S5. An adult-like social-responsive network in young children. (A) Activations obtained for the contrast between social and non-social sentences. The top row shows the joint analysis of all data, while bottom plots show separately the first fMRI (T1, 5-6-year-olds), second fMRI (T2, 6-7-year-olds), and third fMRI (T3, 7-8-year-olds). Voxel-wise $p < 0.001$, FDR-corrected $\alpha < 0.05$. **(B)** Average fMRI signals in social-related ROIs defined in the study presented in S4(*54*). Stars indicate the significance of independent mixed-model regressions conducted at each time step (TR), with condition as fixed effect and participant as random effect (•*p < 0.1; *p < 0.05; **p < 0.01; ***p < 0.001*). Red stars: Math vs. Social. Green stars: Math vs. general knowledge.

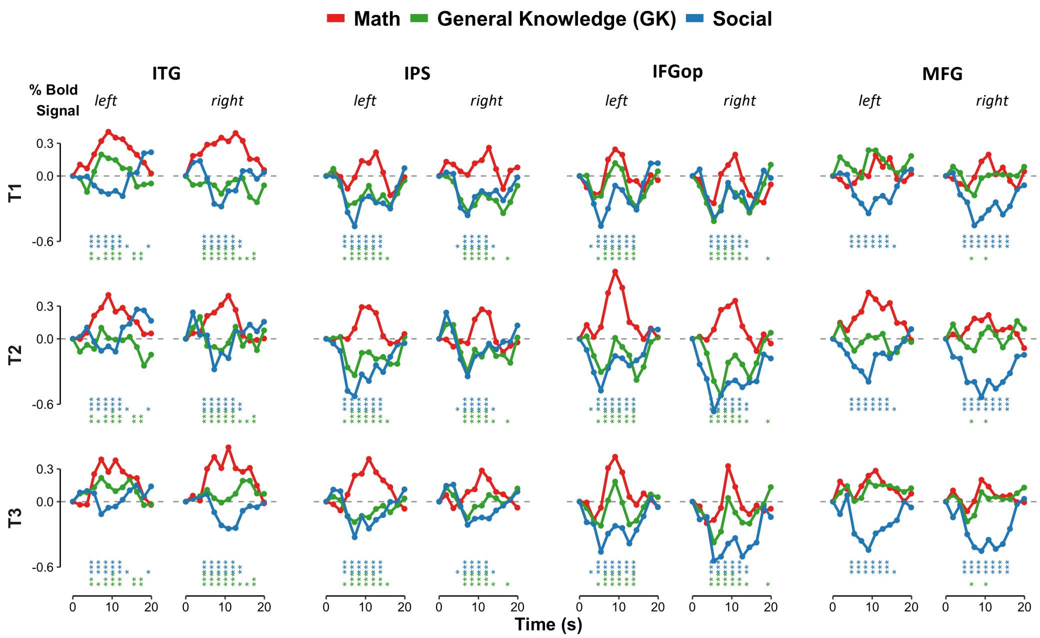

Fig. S6. fMRI signals in adult math-responsive ROIs at each period. Average fMRI signals in math-responsive ROIs defined in the study presented in S4(*54*), at each period (T1, T2, and T3). Stars indicate the significance of independent mixed-model regressions conducted at each time step (TR), with condition as fixed effect and participant as random effect (*•p < 0.1; *p < 0.05; **p < 0.01; ***p < 0.001*). Red stars: Math vs. Social. Green stars: Math vs. general knowledge.

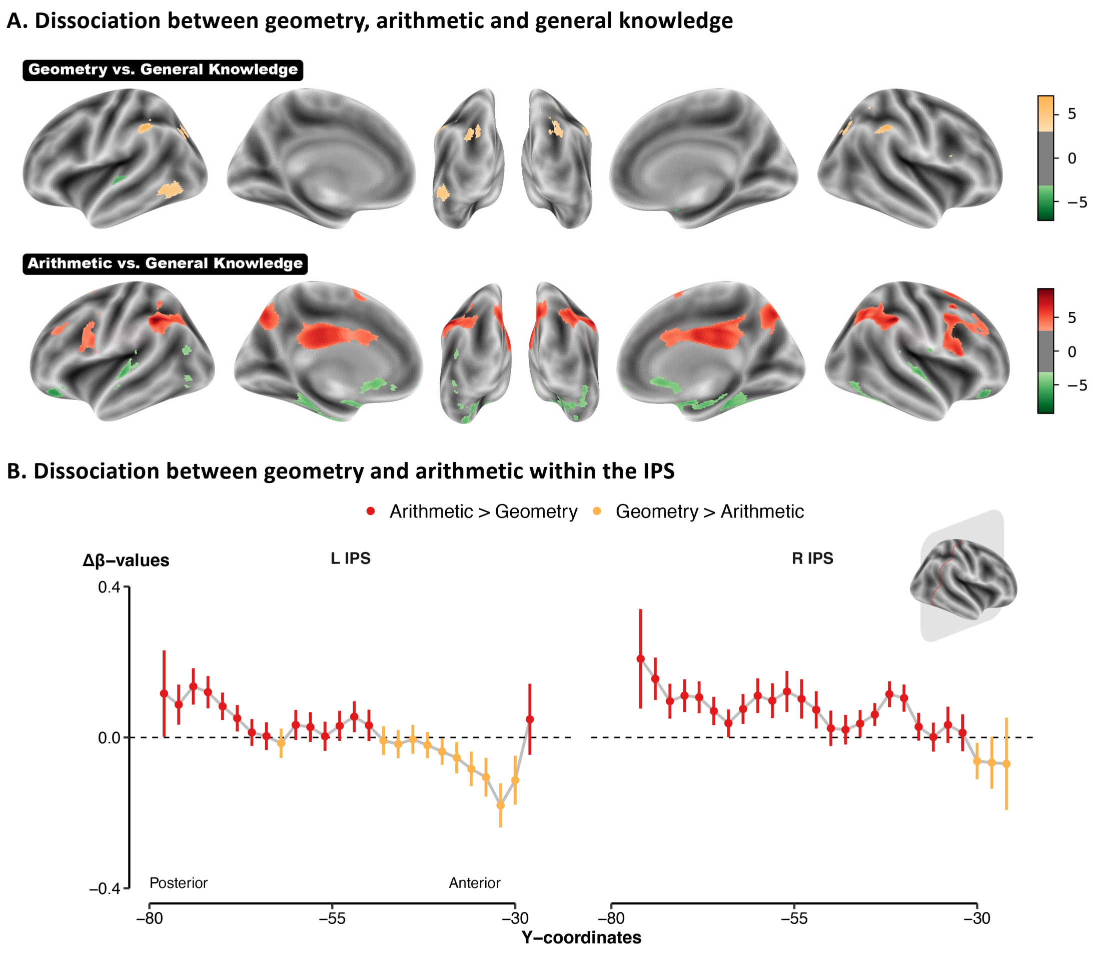

Fig. S7. Geometric and arithmetic activations. (A) Activations obtained for the contrast geometry vs. general knowledge sentences, and arithmetic vs. general knowledge (voxel-wise $p < 0.001$, FDR-corrected $\alpha < 0.05$). **(B)** $\Delta\beta$-values ($\beta_{Arithmetic} - \beta_{Geometry}$) as a function of the y-coordinates in the left and right IPS. Mixed-model regressions with y-coordinates as fixed effect and participant as a random effect.

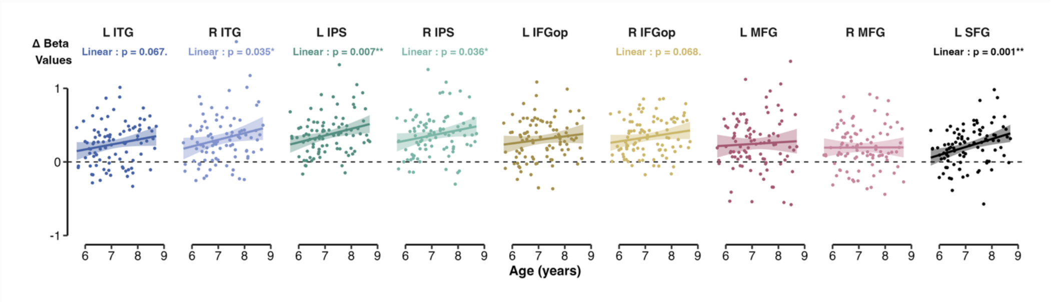

Fig. S8. Development in child-derived ROIs: control analysis for Figure 4A. Each point represents the $\Delta\beta$-values for each participant at each scan and for condition, within each child-derived ROI, as a function of children's chronological age. Mixed-model regressions were conducted within each ROI, using age as fixed effect and participant as a random effect (*$*p < 0.05$; $**p < 0.01$; $***p < 0.001$*).

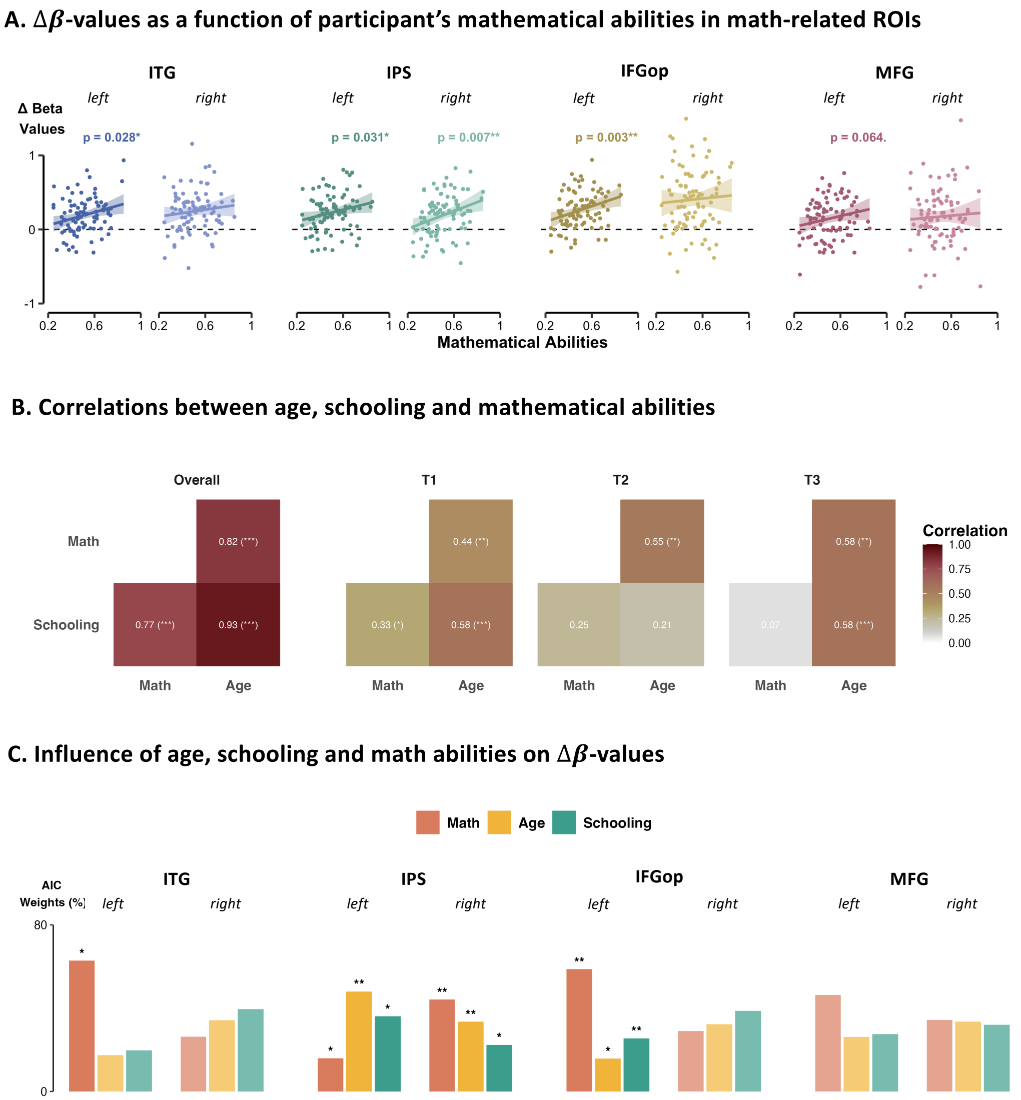

Fig. S9. Partialing out the impact of age, schooling, and individual abilities. (A) Each point represents the $\Delta\beta$-values for each participant at each scan and for condition, within each ROI, as a function of children's mathematical ability. Mixed-model regressions were conducted within each ROI, using individual abilities as fixed effect and participant as a random effect (**p < 0.05; **p < 0.01; ***p < 0.001*). **(B)** Pearson correlations between the three predictors, in each period (T1, T2, T3) and across periods (overall). **(C)** To disentangle the relative influences of these three developmental proxies, we compared a series of mixed models, each including a single predictor as fixed effect, and participant as random effect. Model fit was evaluated using the Akaike information criterion (AIC). To assess the robustness of model differences, we computed the Akaike weights, which estimates the probability that a given model is the best model, considering the evidence from all other models. Akaike weights (%) for each model computed within each ROI are represented here.

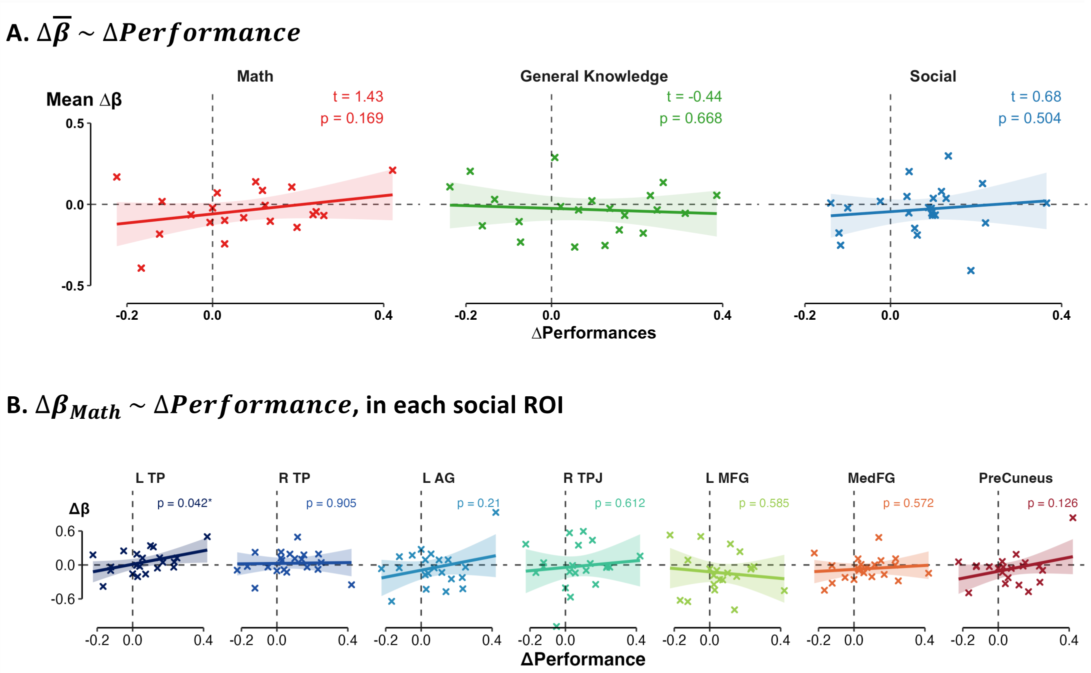

Fig. S10. Control analyses for figure 6, in social-responsive ROIs. Only children scanned both at T1 and T3 (N = 26) were included in the analyses. For each child and sentence, we computed the change in brain activation ($\Delta\beta$-values) by subtracting the β-values at T3 from those at T1, within each social-related ROI. For each sentence, we also computed the average behavioral performance across children at both T1 and T3 and used the difference ($\Delta Perf$) as a proxy for change in comprehension. **(A)** Linear regressions were conducted for each condition, on mean $\Delta\beta$-values averaged across children and social ROIs (*$^*p < 0.05$; $^{**}p < 0.01$; $^{***}p < 0.001$*). **(B)** Linear regressions were performed for only math sentences, within each region-of-interest on mean $\Delta\beta$-values averaged across children (*$^*p < 0.05$; $^{**}p < 0.01$; $^{***}p < 0.001$*).

A. Dimensionality with age in social-related ROIs

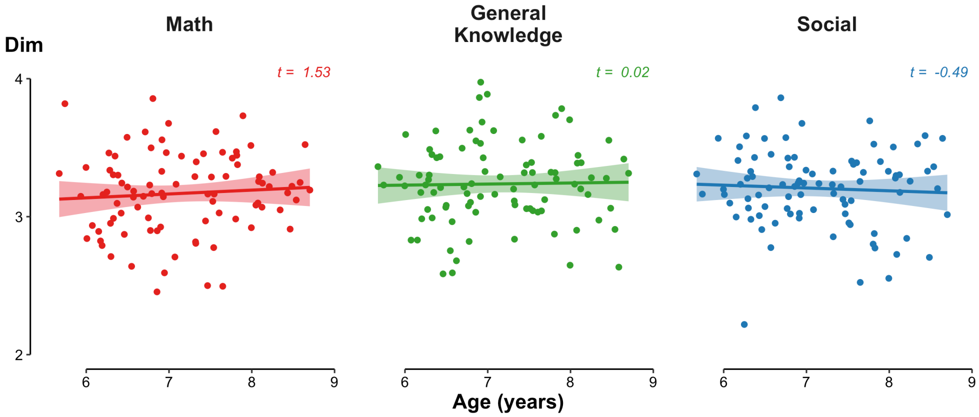

B. Δ-Dimensionality with age in each math-related ROIs

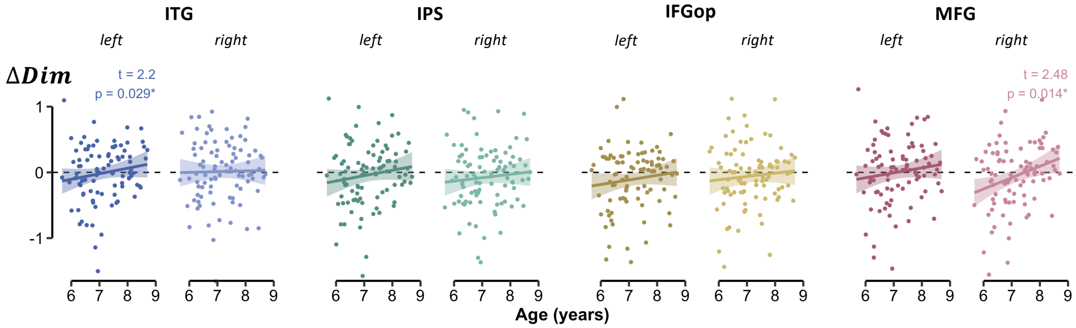

Fig. S11. (A) No increase of neural space for mathematical concepts within social-responsive regions. Each point represents the dimensionality values for each participant and condition, at each scan, averaged across social-responsive ROIs. Mixed model regressions were conducted, using age as fixed effect, and including random intercepts and slopes for age per participant, and a random intercept for ROI (**p < 0.05; **p < 0.01; ***p < 0.001*). **(B)** Each point represents the Δ-dimensionality values (between math and non-math sentences) for each participant, at each scan and within each math-responsive ROI. Mixed model regressions were conducted within each ROI, using age as fixed effect, and participant as random effect (**p < 0.05; **p < 0.01; ***p < 0.001*).

Supplementary Tables

	Estimate	Std. Error	Z-value	P-value
(Intercept)	0.68	0.091	7.34	
**GK vs. Math**	**-0.35**	**0.085**	**-4.08**	**< 0.001 (***)**
**Social vs. Math**	**-0.45**	**0.086**	**-5.29**	**< 0.001 (***)**
**Age**	**-0.24**	**0.061**	**-3.91**	**< 0.001 (***)**
GK vs. Math x Age	0.034	0.084	0.39	0.69
Social vs. Math x Age	0.029	0.085	0.33	0.74

Table S1. Mixed-model coefficients for error rates across conditions. Coefficients obtained from a binomial mixed-model regression on error rates: $Error \sim Condition \times Age + (1|Subject)$, with condition being either math (reference condition), general knowledge or social. The predictor Age was standardized prior to the regression (GK = general knowledge).

	Estimate	Std. Error	DF	t-value	P-value
(Intercept)	1.20	0.028	86.66	43.01	< 0.001 (***)
**GK vs. Math**	**-0.14**	**0.022**	**3904.47**	**-6.63**	**< 0.001 (***)**
**Social vs. Math**	**-0.14**	**0.022**	**3903.95**	**-6.29**	**< 0.001 (***)**
Age	0.014	0.017	3916.90	0.87	0.38
GK vs. Math x Age	-0.013	0.022	3904.23	-0.58	0.57
**Social vs. Math x Age**	**-0.046**	**0.022**	**3903.65**	**-2.13**	**0.034 (*)**

Table S2. Mixed-model coefficients for reaction times across conditions. Coefficients obtained from a linear mixed-model regression on reaction times: $RT \sim Condition \times Age + (1|Subject)$, with condition being either math (reference condition), general knowledge or social. The predictor Age was standardized prior to the regression (GK = general knowledge).

Region	Estimate	Std.	DF	t-value	p-value	p_{FDR}	BF_{10}	%ROPE
Left ITG	-0.083	0.044	317.55	-1.88	0.061	0.163	0.24	65.97
Right ITG	-0.023	0.058	318.20	-0.40	0.692	0.791	0.06	92.14
Left IPS	-0.029	0.046	318.73	-0.63	0.527	0.703	0.06	93.47
Right IPS	0.071	0.047	318.80	1.52	0.129	0.206	0.15	74.82
Left	0.103	0.049	315.68	2.12	0.035 (*)	0.163	0.42	47.49
Right	0.132	0.081	319.37	1.64	0.101	0.202	0.27	35.19
Left MFG	-0.009	0.050	315.89	-0.18	0.858	0.858	0.05	97.70
Right	0.112	0.056	318.1	2.01	0.045 (*)	0.163	0.37	41.59

Table S3. Geometry vs. arithmetic comparisons in math-responsive ROIs. Coefficients obtained from both frequentists mixed-model regressions and Bayesian ones, performed in each mathematical region-of-interest: $\beta \sim \mathrm{Condition} + (1|\mathrm{Subject})$, with condition being either geometry (reference condition) or arithmetic. ROPE = Region of Practical equivalence (% of chance that BF_{10} is within [-0.1,0.1]). High %ROPE values indicate that the effect is likely negligible, whereas low values indicate that a meaningful difference between arithmetic and geometry is plausible.

Although some regions showed uncorrected p-values below .05 (Left IFGop, Right MFG), none of these effects survived FDR correction (all pFDR ≥ .16). Bayesian analyses were consistent with this conclusion: most ROPE percentages exceeded 90%, indicating practically negligible effects. Only left and right IFGop and right MFG showed lower %ROPE values (< 50%), suggesting that small effects cannot be ruled out, although the Bayes Factors indicated only weak evidence.

Region	Predictor	Estimate	Std. Error	DF	t-value	p-value	p_{FDR}
Left ITG	Linear	0.40	0.22	64.03	1.79	0.078	0.16
	Quadratic	-0.038	0.23	72.28	-0.17	0.87	0.99
Right ITG	Linear	0.43	0.26	68.94	1.64	0.105	0.13
	Quadratic	-0.085	0.27	76.19	-0.32	0.75	0.99
**Left IPS**	**Linear**	**0.60**	**0.24**	**72.06**	**2.52**	**0.014 (*)**	**0.037 (*)**
	Quadratic	-0.13	0.24	78.28	-0.53	0.60	0.99
**Right IPS**	**Linear**	**0.67**	**0.24**	**62.80**	**2.83**	**0.006 (**)**	**0.032 (*)**
	Quadratic	-0.099	0.24	71.19	-0.41	0.68	0.99
**Left IFGop**	**Linear**	**0.56**	**0.21**	**59.45**	**2.73**	**0.008 (**)**	**0.032 (*)**
	Quadratic	0.037	0.21	67.57	0.17	0.86	0.99
Right IFGop	Linear	0.36	0.37	67.66	0.97	0.33	0.38
	Quadratic	-0.063	0.37	74.87	-0.17	0.87	0.99
Left MFG	Linear	0.41	0.26	68.93	1.57	0.12	0.16
	Quadratic	0.000	0.26	76.20	-0.002	0.10	0.99
Right MFG	Linear	0.16	0.34	63.60	0.45	0.65	0.65
	Quadratic	0.051	0.35	72.19	0.15	0.88	0.99

Table S4. Mixed-model coefficients for age effects in math-responsive ROIs. Coefficients obtained from mixed-model regressions performed in each mathematical region-of-interest: $\Delta\beta \sim \text{poly}(\text{Age}, 2) + (1|\text{Subject})$, with $\Delta\beta$-values the difference between the activation elicited by mathematical sentences and the mean activation elicited by non-mathematical sentences (general knowledge and social).

Region	Predictor	Estimate	Std.	df	t-value	p-value
Left ITG	Age	0.053	0.030	62.87	1.79	0.079
	Motion	0.054	0.391	89.79	0.14	0.890
Right ITG	Age	0.060	0.034	69.31	1.75	0.085
	Motion	-0.678	0.436	87.84	-1.55	0.124
**Left IPS**	**Age**	**0.082**	**0.032**	**72.22**	**2.58**	**0.012 (*)**
	Motion	-0.372	0.386	80.87	-0.96	0.338
**Right IPS**	**Age**	**0.089**	**0.031**	**63.45**	**2.84**	**0.006 (**)**
	Motion	-0.173	0.419	89.98	-0.41	0.680
**Left IFGop**	**Age**	**0.075**	**0.027**	**61.40**	**2.73**	**0.008 (**)**
	Motion	-0.036	0.377	89.02	-0.10	0.924
Right IFGop	Age	0.051	0.049	68.20	1.03	0.307
	Motion	-0.727	0.647	89.66	-1.12	0.264
Left MFG	Age	0.055	0.035	72.13	1.59	0.117
	Motion	-0.603	0.421	78.19	-1.43	0.157
Right MFG	Age	0.020	0.045	61.93	0.44	0.658
	Motion	0.332	0.587	88.96	0.57	0.572

Supplementary table S5. Controlling for motion effects in developmental changes in math ROIs. Coefficients obtained from mixed-model regressions performed in each mathematical region-of-interest: $\Delta\beta \sim \mathrm{Age} + \mathrm{Motion} + (1|\mathrm{Subject})$, with $\Delta\beta$-values the difference between the activation elicited by mathematical sentences and the mean activation elicited by non-mathematical sentences (general knowledge and social).

Region	Predictor	Estimate	Std. error	df	t-value	p-value
Left ITG	Age	0.052	0.030	62.51	1.77	0.082
	tSNR	0.002	0.003	86.88	0.76	0.452
**Right ITG**	Age	0.055	0.033	70.27	1.65	0.103
	**tSNR**	**0.008**	**0.003**	**83.49**	**2.85**	**0.006 (**)**
**Left IPS**	**Age**	**0.080**	**0.031**	**72.24**	**2.53**	**0.014 (*)**
	tSNR	0.004	0.002	73.98	1.73	0.087
**Right IPS**	**Age**	**0.088**	**0.031**	**63.67**	**2.82**	**0.006 (**)**
	tSNR	0.003	0.003	88.66	1.19	0.237
**Left IFGop**	**Age**	**0.072**	**0.027**	**63.47**	**2.61**	**0.011 (*)**
	tSNR	0.005	0.002	89.51	1.94	0.056
Right IFGop	Age	0.045	0.049	69.20	0.93	0.358
	tSNR	0.008	0.004	87.55	1.98	0.051
Left MFG	Age	0.052	0.034	90.00	1.51	0.135
	tSNR	0.006	0.003	90.00	2.19	0.031
Right MFG	Age	0.022	0.045	62.62	0.49	0.629
	tSNR	-	0.004	86.30	-	0.608

Table S6. Controlling for noise effects in developmental changes in math ROIs. Coefficients obtained from mixed-model regressions performed in each mathematical region-of-interest: $\Delta\beta \sim \text{Age} + \text{tSNR} + (1|\text{Subject})$, with $\Delta\beta$-values the difference between the activation elicited by mathematical sentences and the mean activation elicited by non-mathematical sentences (general knowledge and social).

Region	Estimate	Std. Error	DF	t-value	p-value	p_{FDR}
**Left ITG**	**0.387**	**0.173**	**76.36**	**2.24**	**0.028 (*)**	**0.062**
Right ITG	0.223	0.202	81.39	1.10	0.273	0.364
**Left IPS**	**0.403**	**0.184**	**85.92**	**2.19**	**0.031 (*)**	**0.062**
**Right IPS**	**0.521**	**0.188**	**73.66**	**2.77**	**0.007 (**)**	**0.028**
**Left IFGop**	**0.505**	**0.165**	**72.08**	**3.06**	**0.003 (**)**	**0.024**
Right IFGop	0.167	0.291	76.59	0.57	0.569	0.650
Left MFG	0.375	0.200	86.02	1.88	0.064	0.102
Right MFG	0.119	0.271	79.28	0.44	0.662	0.662

Table S7. Math-ability effects in math-responsive ROIs. Coefficients obtained from mixed-model regressions performed in each mathematical region-of-interest: $\Delta\beta \sim \mathrm{Math}$, with $\Delta\beta$-values the difference between the activation elicited by mathematical sentences and the mean activation elicited by non-mathematical sentences (general knowledge and social), and math the individual mathematical abilities obtained from the number screener test.

Region	Estimate	Std. Error	t	p-value	p_{FDR}
Left ITG	1.70	2.32	0.73	0.469	0.536
Right ITG	6.52	2.94	2.22	0.0313 (*)	0.0682
Left IPS	5.95	2.04	2.92	0.0052 (**)	0.0418 (*)
Right IPS	4.98	2.28	2.18	0.0341 (*)	0.0682
Left IFGop	6.13	2.34	2.62	0.0115 (*)	0.0461 (*)
Right IFGop	4.73	4.60	1.03	0.309	0.412
Left MFG	4.65	2.38	1.96	0.0558	0.0893
Right MFG	0.36	3.30	0.11	0.913	0.913

Table S8. Amplified vs. reduced voxels percentages in math-responsive ROIs. Coefficients obtained from linear regressions performed in each mathematical region-of-interest: $\text{Percentages} \sim \text{Category}$, comparing the percentages of amplified voxels ($\Delta\beta(T_3) > \Delta\beta(T_1), and\ \Delta\beta(T_1) > 0$) vs. reduced voxels ($\Delta\beta(T_3) < \Delta\beta(T_1), and\ \Delta\beta(T_1) > 0$). Degree of freedom = 25.

Region	Estimate	Std. Error	t	p-Value	p_{FDR}
Left ITG	5.06	2.18	2.32	0.0245 (*)	0.0655
Right ITG	1.00	2.87	0.35	0.728	0.728
Left IPS	6.40	2.52	2.54	0.0141 (*)	0.0564
Right IPS	4.93	2.27	2.17	0.0348 (*)	0.0696
Left IFGop	7.43	2.16	3.45	0.0012 (**)	0.0093 (**)
Right IFGop	2.14	4.01	0.53	0.595	0.681
Left MFG	5.42	2.92	1.85	0.0696	0.111
Right MFG	3.31	3.59	0.92	0.362	0.482

Table S9. Recruited vs. dropout voxel percentages in math-responsive ROIs. Coefficients obtained from linear regressions performed in each mathematical region-of-interest: $\text{Percentages} \sim \text{Category}$, comparing the percentage of recruited voxels ($\Delta\beta(T_3) > 0, and\ \Delta\beta(T_1) < 0$) vs. dropout voxels ($\Delta\beta(T_3) < 0, and\ \Delta\beta(T_1) > 0$). Degree of freedom = 25.

Region	Estimate	Std. Error	DF	t-value	p-value	p_{FDR}
Left ITG	-0.93	0.39	19	-2.35	0.030 (*)	0.06
Right ITG	-1.36	0.34	19	-4.04	0.001 (***)	0.008 (***)
Left IPS	-0.36	0.57	19	-0.63	0.53	0.61
Right IPS	-0.20	0.48	19	-0.41	0.69	0.69
Left IFGop	-1.01	0.42	19	-2.38	0.028 (*)	0.06
Right IFGop	-1.13	0.58	19	-1.95	0.066	0.11
Left MFG	-0.69	0.49	19	-1.41	0.17	0.23
Right MFG	-1.06	0.40	19	-2.73	0.013 (*)	0.05

Table S10. Brain–performance change relationships in math-responsive ROIs for math sentences. Coefficients obtained from linear regressions performed in each mathematical region-of-interest: $\Delta\beta \sim \Delta\mathrm{Perf}$, with each data point corresponding to an individual mathematical sentence. $\Delta\beta$-values correspond to the difference in brain activation between T3 and T1, elicited by each sentence and averaged across participants. $\Delta\mathrm{Perf}$ represents the change in behavioral accuracy between T3 and T1 for each sentence, averaged across participants.

Region	Estimate	Std. Error	DF	t-value	p-value	p_{FDR}
Left TP	0.60	0.27	19	2.18	0.042 (*)	0.29
Right TP	0.037	0.30	19	0.12	0.90	0.90
Left AG	0.61	0.47	19	1.30	0.21	0.49
Right TPJ	0.29	0.57	19	0.51	0.61	0.71
Left MFG	-0.29	0.52	19	-0.55	0.58	0.71
MedFG	0.17	0.30	19	0.57	0.57	0.71
Precuneus	0.61	0.38	19	1.60	0.13	0.44

Table S11. Brain–performance change relationships in social-responsive ROIs for math sentences. Coefficients obtained from linear regressions performed in each social region-of-interest: $\Delta\beta \sim \Delta\mathrm{Perf}$, with each data point corresponding to an individual mathematical sentence. $\Delta\beta$-values correspond to the difference in brain activation at T3 minus T1, elicited by each sentence and averaged across participants. $\Delta\mathrm{Perf}$ is the difference between average performance at T3 minus T1 for each sentence, averaged across participants.

Region	Estimate	Std. Error	DF	t-value	p-value	p_{FDR}
Left ITG	0.12	0.05	89	2.20	.029 (*)	0.12
Right ITG	-0.01	0.06	89	-0.19	.85	0.85
Left IPS	0.07	0.05	89	1.40	.16	0.44
Right IPS	0.06	0.06	89	1.04	.30	0.48
Left IFGop	0.04	0.05	89	0.70	.49	0.55
Right IFGop	0.05	0.05	89	0.88	.38	0.50
Left MFG	0.06	0.06	89	1.11	.27	0.48
Right MFG	0.14	0.06	89	2.48	.014 (*)	0.11

Table S12. Dimensionality changes in math-responsive ROIs. Coefficients obtained from linear regressions performed in each math-responsive ROI: $\Delta\mathrm{Dim} \sim \mathrm{Age}$. $\Delta\mathrm{Dim}$-values correspond to the difference between the dimensionality for mathematical sentences and the mean dimensionality for non-mathematical sentences (general knowledge and social).
